# Targeting estrogen-regulated system x_c_^−^ promotes ferroptosis and endocrine sensitivity of ER+ breast cancer

**DOI:** 10.1038/s41419-025-07354-0

**Published:** 2025-01-20

**Authors:** Jiawei Cao, Tong Zhou, Tao Wu, Rixu Lin, Ju Huang, Dejin Shi, Jiawei Yu, Yinrui Ren, Changrui Qian, Licai He, Guang Wu, Zhixiong Dong, Shaofei Yuan, Haihua Gu

**Affiliations:** 1 Wenzhou Key Laboratory of Cancer Pathogenesis and Translation, Key Laboratory of Laboratory Medicine, School of Laboratory Medicine and Life Sciences, Ministry of Education, Wenzhou Medical University, 325035 Wenzhou, China; 2 Department of Pathology, The First Affiliated Hospital of Wenzhou Medical University, 325035 Wenzhou, China; 3 School of Basic Medical Sciences, Wenzhou Medical University, 325035 Wenzhou, China; 4 Department of Medical Oncology, Rui’an People’s Hospital, The Third Affiliated Hospital of Wenzhou Medical University, 325200 Wenzhou, China

**Keywords:** Breast cancer, Cell death, Cell growth

## Abstract

Estrogen receptor positive (ER+) breast cancer accounts for approximately 70% of cases. Endocrine therapies targeting estrogen are the first line therapies for ER+ breast cancer. However, resistance to these therapies occurs in about half of patients, leading to decreased survival rates. Inducing ferroptosis is a promising therapeutic strategy for cancer treatment for refractory and malignant cancers including triple-negative breast cancer. Nevertheless, ER+ breast cancer is relatively resistant to ferroptosis inducers. Here, we uncovered that ERα suppressed ferroptosis in ER+ breast cancer. Silencing ERα triggered ferroptosis, which was attenuated by ferroptosis inhibitor Ferrostatin-1, and was enhanced by ferroptosis inducer Erastin. Mechanistically, ERα transcriptionally upregulated the expression of SLC7A11 and SLC3A2, two subunits of the system x_c_^−^, which is one key inhibitory regulator of ferroptosis. Overexpression of the exogenous SLC7A11 and SLC3A2 was able to mitigate ferroptosis induced by ERα inhibition. Moreover, SLC7A11 and SLC3A2 levels were elevated in endocrine-resistant breast cancer cells and tumors. Importantly, the system x_c_^−^ inhibitor Sorafenib or Imidazole ketone erastin effectively inhibited the growth of tamoxifen-resistant breast cells in vitro and in vivo. In conclusion, our data reveal that targeting estrogen-regulated SLC7A11 and SLC3A2 enhances ferroptosis in ER+ breast cancer, offering a novel therapeutic option for patients with ER+ breast cancer, particularly those with endocrine resistance.

## Introduction

Surpassing lung cancer, breast cancer has emerged as the foremost type of cancer globally [1]. Estrogen receptor positive (ER+) breast cancer constitutes approximately 70% of all breast cancer cases, relying on estrogen for its growth [2, 3]. ERα, a steroid hormone receptor processing a multifunctional domain, serves as the principal driver of ER+ breast cancer by acting primarily as a transcription factor. Upon binding to estrogen, ERα translocates to the nucleus where it binds to DNA regions containing estrogen-responsive elements (ERE) and subsequently regulates the transcription of numerous genes that are crucial for breast tumorigenesis [4]. Anti-estrogen-based endocrine therapies, such as the ERα modulator Tamoxifen, ERα degrader Fulvestrant, and the aromatase inhibitor Letrozole, have significantly enhanced the survival rates of patients with hormone receptor-positive (HR+) breast cancer [5]. However, approximately half of the patients undergoing endocrine therapy suffer relapses, including mutations in the ERα coding gene *ESR1*, especially the L536Q and Y537S mutations [6], which present a significant clinical challenge.

Ferroptosis, a form of regulated cell death identified in 2012 [7], differs from other forms of regulated cell death such as apoptosis and pyroptosis, as well as necrosis. Ferroptosis occurs due to its iron-dependent catalysis of lipid peroxidation, leading to the accumulation of lipid peroxides (lipid ROS) in cell membranes and the subsequent cell demise [8, 9]. System x_c_^−^-GPX4 axis plays a critical role in regulating ferroptosis by maintaining cellular redox homeostasis and alleviating oxidative stress. System x_c_^−^ functions as an amino acid antiporter that consists of the light chain SLC7A11 (the amino acid transporter solute carrier family 7 member) and the heavy chain SLC3A2. SLC7A11 acts as an exchange transporter to import cystine in exchange for glutamate, whereas SLC3A2 functions as a molecular chaperone to maintain SLC7A11 protein stability and membrane localization [10]. Cystine is a crucial precursor for the biosynthesis of glutathione (GSH), a critical antioxidant facilitating the elimination of free lipid ROS [7, 11]. Glutathione peroxidase 4 (GPX4) reduces cytotoxic lipid ROS through converting GSH into oxidized glutathione (GSSG), resulting in the inhibition of ferroptosis [12]. System x_c_^−^ mediated uptake of extracellular cystine serves as the principal method by which many cancer cells acquire cellular cystine [10]. Drugs including Erastin and sorafenib, which induce ferroptosis by inhibiting the system x_c_^−^, are promising anticancer therapeutics [13, 14].

Ferroptosis plays an important role in breast cancer, especially in triple-negative breast cancer (TNBC). Fang et al. demonstrated that the luminal androgen receptor (LAR) subtype of TNBC is characterized by the upregulation of glutathione metabolism, which allowed the utilization of Glutathione peroxidase 4 (GPX4) inhibitors to induce ferroptosis of TNBC [15]. Tumor-associated macrophages can secrete transforming growth factor beta (TGF-β) to turn on hepatic leukemia factor (HLF), which enhances the transcription of gamma-glutamyltransferase 1 (GGT1) and promotes resistance to ferroptosis in TNBC [16]. BRD4 inhibitor (+)-JQ-1 decreases the expression of ferroptosis-associated genes and induces ferroptosis of TNBC cells [17]. Compared to TNBC, the regulation of ferroptosis in ER+ breast cancer is still largely unknown. Interestingly, ER+ breast cancer cells are less sensitive to ferroptosis inducers than TNBC cells [18]. Importantly, how ER+ breast cancer cells acquire resistance to ferroptotic insults and whether estrogen plays any role in regulating ferroptosis in ER+ breast cancer remain poorly understood.

Our research revealed that estrogen suppresses ferroptosis in ER+ breast cancer by increasing the expression of SLC7A11 and SLC3A2. ERα interacts with the estrogen response elements (EREs) in the promoter region of *SLC7A11* and *SLC3A2*, thereby enhancing their transcription. Additionally, SLC7A11 and SLC3A2 were found to be upregulated in cells resistant to endocrine therapy. Targeting ferroptosis could potentially be a viable therapeutic approach for ER+ breast cancer, particularly in the cases of endocrine resistance.

## Results

### Estrogen/ERα inhibits ferroptosis of ER+ breast cancer cells

**Fig. 1:**
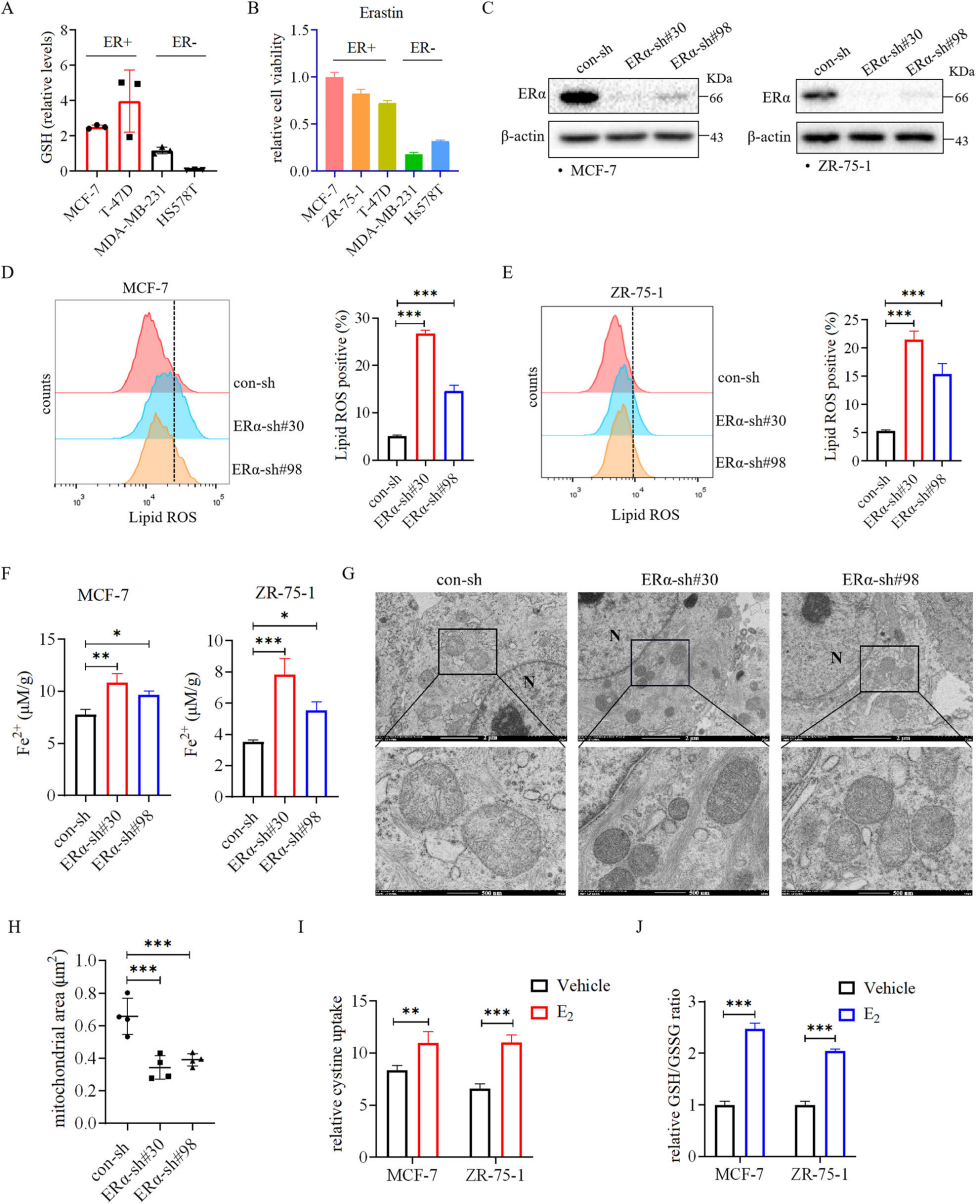
Estrogen/ERα is involved in regulating ferroptosis in ER+ breast cancer cells. **A** Glutathione levels were significantly higher in ER+ breast cancer cell lines compared with in ER- breast cancer cell lines after analyzing the NCI-60 metabolomics dataset. **B** ER- breast cancer cells were more sensitive to the ferroptosis inducer Erastin than ER+ breast cancer cells. Cells were treated with Erastin (10 μM) for 72 h before being analyzed by the CCK8 reagent. **C** ERα was effectively knocked down using shRNA in ER+ breast cancer cell lines MCF-7 and ZR-75-1. Cells expressing two different ERα shRNAs (sh30 and sh98) were subjected to western blot analysis. **D**, **E** Knockdown of ERα increased Lipid ROS levels in ER+ breast cancer cells. Con- and ERα-knocked down MCF-7 (**D**) and ZR-75-1 (**E**) cells were treated with BODIPY 581/591C11 (10 μM) dye for 30 min. Flow cytometry was used to detect Lipid ROS levels. **F** Knockdown of ERα increased Fe^2+^ levels in ER+ breast cancer cells. **G**, **H** Knockdown of ERα decreased the size of mitochondria in MCF-7 cells. Transmission electron microscopy revealed a distinctive morphological feature of ferroptosis in ERα-knocked down MCF-7 cells (**G**). Quantitation of the mitochondrial area in con- and ERα-knocked down MCF-7 cells was presented, *n* = 4 in each group (**H**). **I**, **J** Estrogen enhanced antioxidant capacity of ER+ breast cancer cells. Estrogen starved MCF-7 and ZR-75-1 cells were treated with or without estrogen (E_2_) (10 nM) for 24 h before being subjected to analysis for cystine uptake (I) and the ratio of glutathione (GSH) to oxidized glutathione (GSSG) (**J**). Data are shown as Mean ± SEM (*n* = 3). *P < 0.05, **P < 0.01, ***P < 0.001.

To explore the role of estrogen and ERα in regulating ferroptosis in ER+ breast cancer, we first compared the levels of glutathione, a key antioxidant against ferroptosis, in ER+ and ER- breast cancer cell lines. Previous studies have indicated a notable disparity in GSH levels between ER+ and ER- breast tumors, with the former exhibiting significantly elevated expression of GSH [19]. To further investigate this phenomenon, we conducted an analysis of GSH levels in various breast cancer cell lines utilizing the NCI-60 metabolomics dataset. Our analysis revealed that GSH levels were significantly higher in ER+ breast cancer cells compared to ER- breast cancer cells (Fig. 1A), mirroring the trends observed in clinical samples [19]. Subsequently, we found that ER- breast cancer cell lines exhibited greater susceptibility to the ferroptosis inducer Erastin compared to ER+ breast cancer cell lines (Fig. 1B). Next, we investigated whether ERα is involved in regulating ferroptosis by silencing ERα in ER+ breast cancer cells (Fig. 1C). Our result revealed that ERα knockdown elevated the levels of Lipid ROS (Fig. 1D, E) and Fe^2+^ content (Fig. 1F) within the cells. Additionally, ERα knockdown led to morphological changes in mitochondria, including shrinkage and increased density (Fig. 1G), as well as a reduction in mitochondrial area (Fig. 1H), which were consistent with the morphological changes of cells undergoing ferroptosis [20]. Conversely, estrogen stimulation enhanced cystine uptake (Fig. 1I) and the ratio of GSH/GSSG (Fig. 1J) in ER+ breast cancer cell lines.

**Fig. 2:**
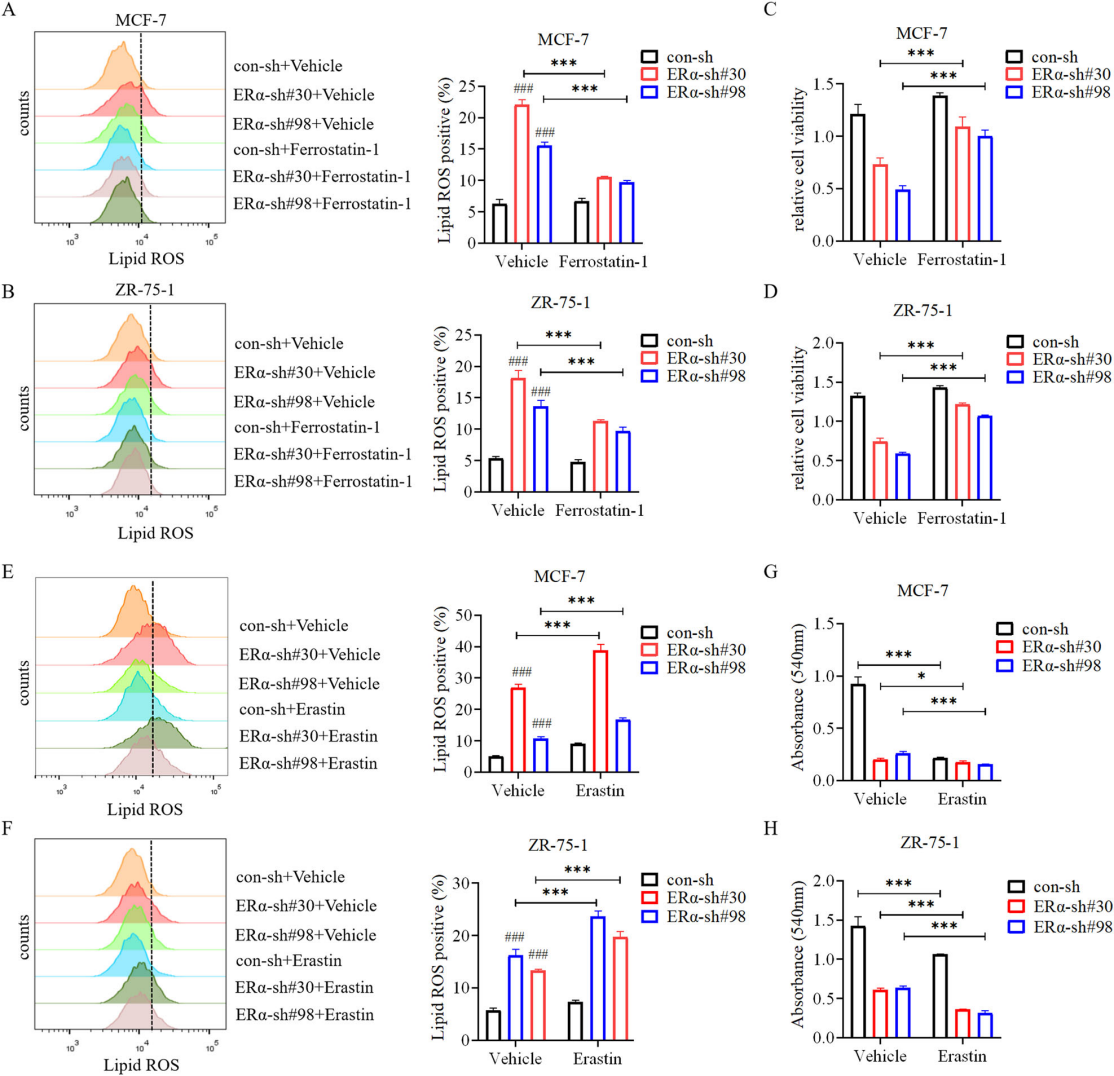
Ferroptosis inhibitor Ferrostatin-1 attenuates, whereas Erastin enhances ferroptosis induced by knockdown of ERα. **A**, **B** Ferrostatin-1 partially inhibited cell ferroptosis caused by ERα knockdown. ERα-knocked down MCF-7 (**A**) and ZR-75-1 (**B**) cells were treated with vehicle or Ferrostatin-1 (5 μM) alone for 24 h. Lipid ROS were detected by flow cytometry. **C**, **D** Ferrostatin-1 treatment rescued the decrease in cell growth induced by ERα knockdown in MCF-7 (**C**) and ZR-75-1 (**D**) cells. Cells were treated with Ferrostatin-1 (5 μM) for 72 h before being analyzed using the CCK8 reagent. **E**, **F** The ferroptosis inducer Erastin enhanced cell ferroptosis induced by ERα knockdown. ERα-knocked down MCF-7 (**E**) and ZR-75-1 (**F**) cells were treated with vehicle and Erastin (10 μM) alone for 24 h. Lipid ROS were detected by flow cytometry. **G**, **H** Erastin treatment enhanced the decrease in cell growth induced by ERα knockdown. MCF-7 (**G**) and ZR-75-1 (**H**) cells were treated with Erastin (10 μM) for 72 h before being analyzed using the CCK8 reagent. Data are shown as Mean ± SEM (*n* = 3). *P < 0.05, ***P < 0.001, ^###^*P* < 0.001.

To further support that ERα knockdown induced ferroptosis, ER+ breast cancer cells were exposed to the ferroptosis inhibitor Ferrostatin-1. Ferrostatin-1 partially attenuated the increase in Lipid ROS resulting from ERα knockdown (Fig. 2A, B), as well as partially rescued the decrease in cell viability caused by ERα knockdown (Fig. 2C, D). Additionally, ferroptosis inducer Erastin exacerbated Lipid ROS levels (Fig. 2E, F) and growth inhibition (Fig. 2G, H) induced by ERα knockdown. These results indicated that ERα plays an important role in suppressing ferroptosis in ER+ breast cancer cells.

### Expression of system x_c_^−^ was positively correlated with ERα expression, and predicted the poor outcome of ER+ breast cancer

**Fig. 3:**
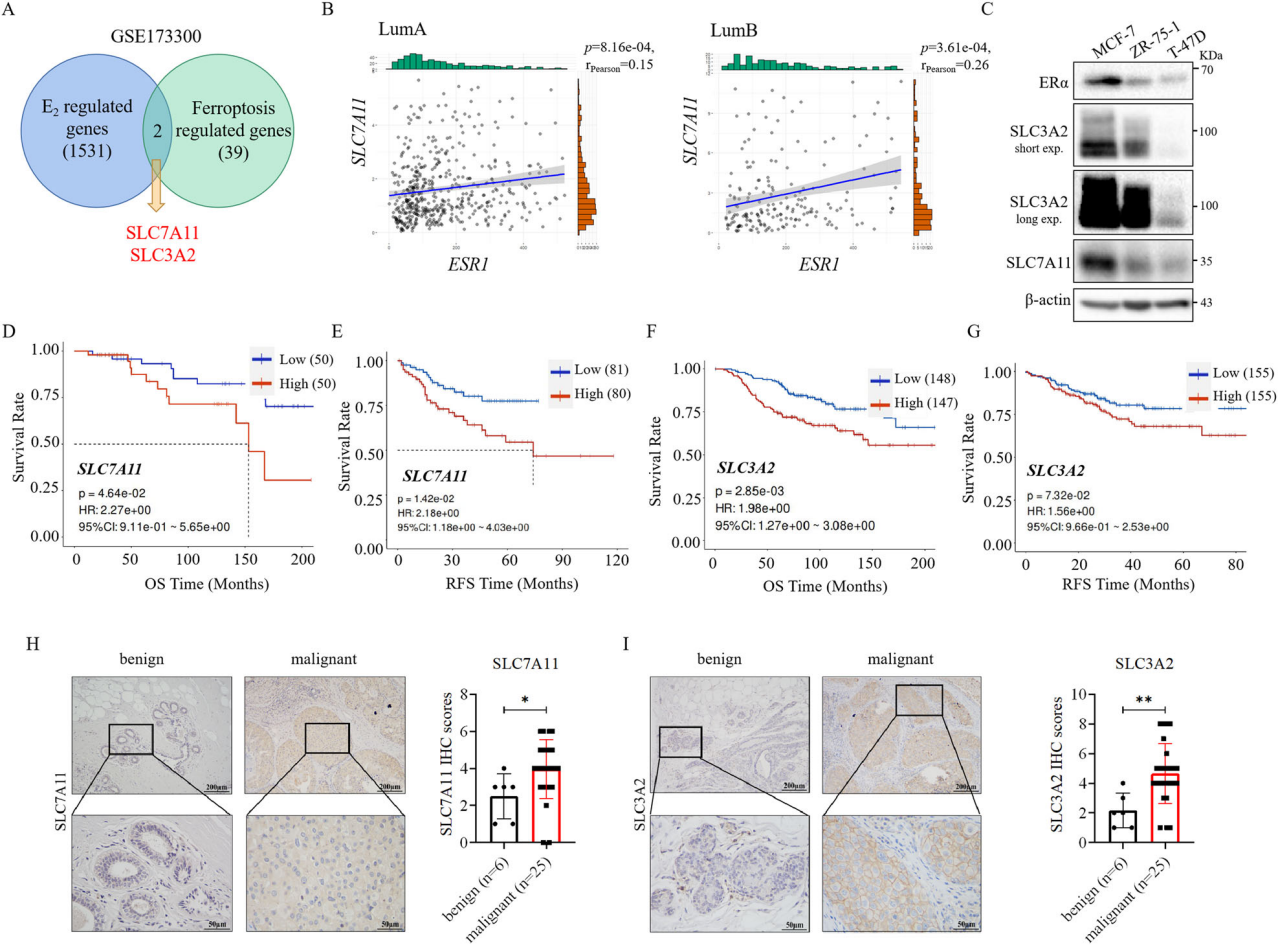
High level of SLC7A11/SLC3A2 is positively correlated with ERα and poor prognosis in breast cancer. **A** Analysis of the GEO transcriptome dataset GSE173300 showed an overlap of 2 genes between E_2_-regulated genes and ferroptosis-regulated genes in MCF-7 cells. **B** Analysis of the TCGA database showed a positive correlation between *SLC7A11* and *ESR1* mRNA levels in Luminal A and Luminal B breast cancer. **C** Western blot analysis showed SLC3A2 and SLC7A11 protein levels in ER+ breast cancer cell lines MCF-7, ZR-75-1, and T47D. **D**–**G** Analysis of the GEO databases showed that breast cancer patients with higher SLC7A11 (**D**, **E**) (GSE26304, GSE18229) and SLC3A2 (**F**, **G**) (GSE159956, GSE25055) expression levels had shorter overall survival (**D**, **F**) and relapse-free survival times (**E**, **G**). **H**, **I**, Immunohistochemistry (IHC) analysis showed that SLC7A11 (**H**) and SLC3A2 (**I**) protein levels were significantly higher in malignant breast tumors compared with benign breast tissues. Scale bars, 200 μm and 50 μm. SLC7A11 and SLC3A2 IHC staining were scored in breast tumor tissues from 31 patients. *P < 0.05, **P < 0.01.

To investigate how ERα regulates ferroptosis in ER+ breast cancer cells, we conducted an analysis of ferroptosis-related genes that are also regulated by estrogen. Examination of the GEO dataset of estrogen-stimulated MCF-7 cells revealed that the expression of *SLC7A11* and *SLC3A2*, components of the system x_c_^−^ responsible for cystine uptake, was upregulated following estrogen treatment (Fig. 3A, S1). Furthermore, analysis of the TCGA dataset indicated a weak positive correlation between the mRNA levels of *SLC7A11* and *ESR1* in luminal A and luminal B subtypes of breast cancer (Fig. 3B). Additionally, the protein levels of SLC7A11 and SLC3A2 were found to be positively correlated with ERα protein levels in ER+ breast cancer cell lines (Fig. 3C). Moreover, elevated mRNAs levels of *SLC7A11* and *SLC3A2* were correlated to decreased overall survival and relapse-free survival rates of breast cancer (Fig. 3D–G). Importantly, malignant breast cancer tissues exhibited higher protein levels of SLC7A11 and SLC3A2 compared to benign breast tissues (Fig. 3H, I), indicating a positive association of SLC7A11 and SLC3A2 levels with poor prognosis in breast cancer patients. These findings revealed a positive correlation between the expression of SLC7A11 and SLC3A2 with ERα expression, further implicating their role in the adverse outcomes of breast cancer.

### ERα transcriptionally promotes the expression of SLC7A11 and SLC3A2

**Fig. 4:**
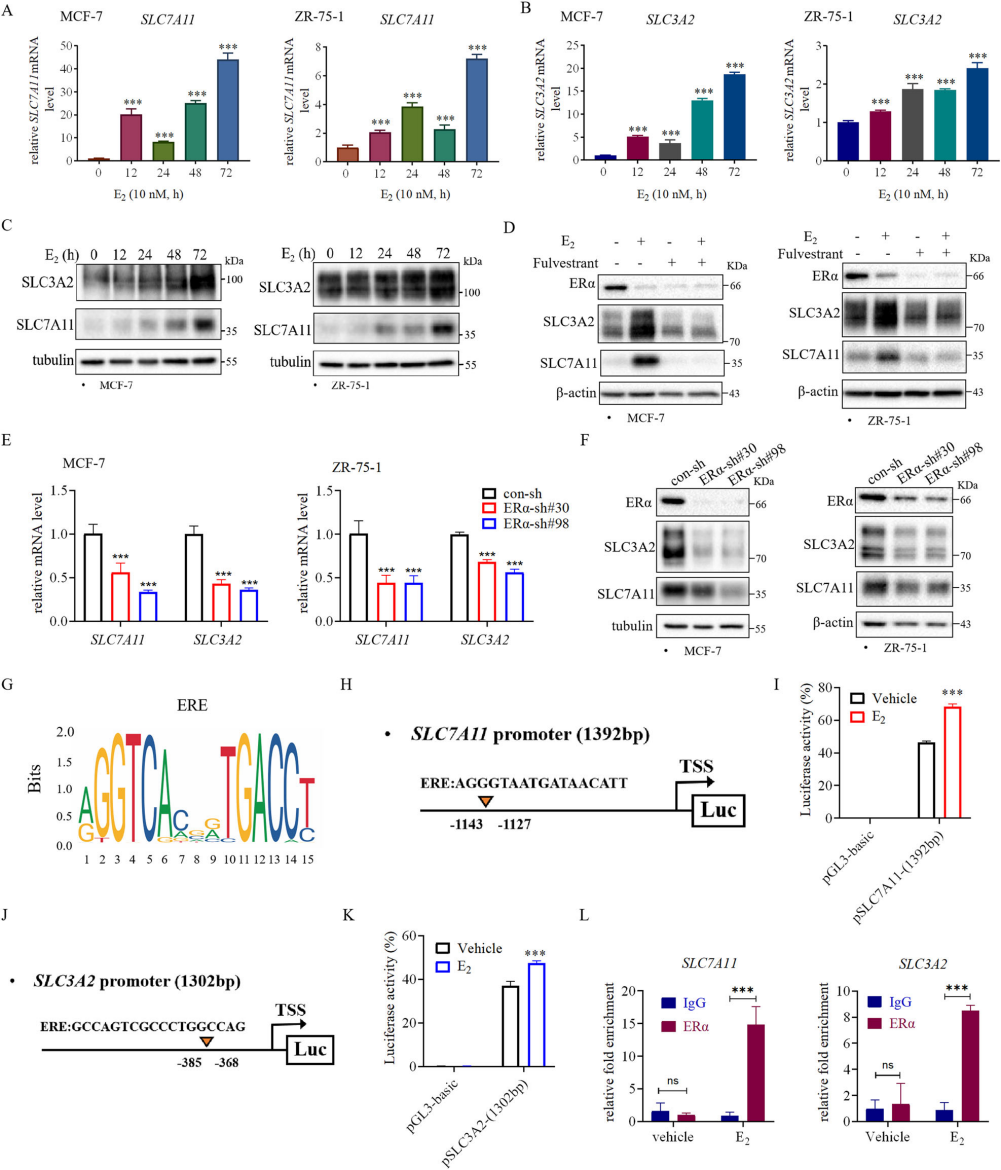
ERα transcriptionally promotes the expression of SLC7A11 and SLC3A2 in ER+ breast cancer cells. Estrogen (E_2_) increases *SLC7A11* (**A**) and *SLC3A2* (**B**) mRNA levels in ER+ breast cancer cell lines. *SLC7A11* (**A**) and *SLC3A2* (**B**) mRNA levels were quantified by qRT-PCR in estrogen-starved MCF-7 and ZR-75-1 cells treated with 10 nM E_2_ for 0, 12, 24, 48, and 72 h. **C** E_2_ increases SLC7A11 and SLC3A2 protein levels in ER+ breast cancer cell lines. SLC7A11 and SLC3A2 protein levels were examined by western blot analysis in MCF-7 and ZR-75-1 cells treated with 10 nM E_2_ for 0, 12, 24, 48, and 72 h. **D** Fulvestrant prevented the increase in SLC7A11 and SLC3A2 protein levels enhanced by E_2_ treatment. Estrogen-starved MCF-7 and ZR-75-1 cells were pretreated with 1 µM Fulvestrant for 2 h before the addition of 10 nM E_2_ for 72 h followed by western blot analysis for detecting ERα, SLC7A11, and SLC3A2 protein levels. **E** Knockdown of ERα decreased the mRNA levels of *SLC7A11* and *SLC3A2*. *SLC7A11* and *SLC3A2* mRNA levels were quantified by qRT-PCR in con- and ERα-knocked down MCF-7 and ZR-75-1 cells. **F** Knockdown of ERα reduced the protein levels of SLC7A11 and SLC3A2 in MCF-7 and ZR-75-1 cells. **G** The motif of Estrogen-responsive element (*ERE*) as predicted by the JASPAR website. **H**, **J** pGL3-*SLC7A11* and pGL3-*SLC3A2* promoter luciferase reporter constructs. One predicted *ERE* in the SLC7A11 promoter region, and one predicted *ERE* in the *SLC3A2* promoter region were indicated by arrowheads. **I**, **K** Luciferase reporter assay showed that E_2_ enhanced *SLC7A11* (**I**) or *SLC3A2* (**K**) promoter activity in MCF-7 cells. The indicated luciferase reporter plasmids together with the TK-renilla luciferase plasmid were transiently transfected into MCF-7 cells. Cells were treated with 10 nM E_2_ for 24 h before being subjected to luciferase activity assay. **L** Estrogen promoted ERα binding to the *SLC7A11* or *SLC3A2* promoter region containing the predicted ERE in MCF-7 cells. The binding of ERα to the *SLC7A11/ SLC3A2* promoter regions was analyzed using the ERα-ChIP experiments. Data are shown as Mean ± SEM (*n* = 3). ***P < 0.001; ns not significant.

Given the observed association between ERα and SLC7A11/SLC3A2 expression, we investigated how ERα may regulate the expression SLC7A11 and SLC3A2. q-PCR analysis demonstrated that estrogen significantly increased the mRNA levels of *SLC7A11* and *SLC3A2* in ER+ breast cancer cells (Fig. 4A, B). Accordingly, estrogen treatment also elevated the protein levels of SLC7A11 and SLC3A2 (Fig. 4C). In contrast, treatment with Fulvestrant, an ERα degradation drug, effectively inhibited estrogen-induced protein levels of SLC7A11 and SLC3A2 (Fig. 4D). Accordingly, knockdown of ERα resulted in decreased levels of both SLC7A11 and SLC3A2 mRNA and protein (Fig. 4E, F).

We further investigated whether ERα acts as a transcription factor to directly regulate the transcription of *SLC7A11* and *SLC3A2*. Analysis of the promoter regions of *SLC7A11* and *SLC3A2* utilizing the online resource JASPAR (http://jaspar.genereg.net/) revealed the presence of potential estrogen response element (EREs), resembling the ERα binding motif, situated within the 1.5 kb promoter regions upstream of the transcription start sites of *SLC7A11* and *SLC3A2* (Fig. 4G–J). Luciferase reporter experiment showed that these *SLC7A11* and *SLC3A2* promoter DNA fragments were able to confer estrogen-induced luciferase activities in MCF-7 cells (Fig. 4I, K). In addition, chromatin immunoprecipitation (ChIP) assay was conducted to investigate ERα binding to the predicted ERE-containing promoter regions in *SLC7A11* and *SLC3A2*. In estrogen-starved conditions, ERα exhibited no basal binding, but upon estrogen stimulation, ERα was found to bind to the predicted ERE-containing regions in *SLC7A11* and *SLC3A2* (Fig. 4L). Together, these results indicate that ERα is recruited to the ERE-containing promoters in *SLC7A11* and *SLC3A2*, thereby enhancing SLC7A11 and SLC3A2 expression in response to estrogen stimulation in ER+ breast cancer cells.

### SLC7A11 and SLC3A2 mediate ERα inhibition of ferroptosis in ER+ breast cancer cells

**Fig. 5:**
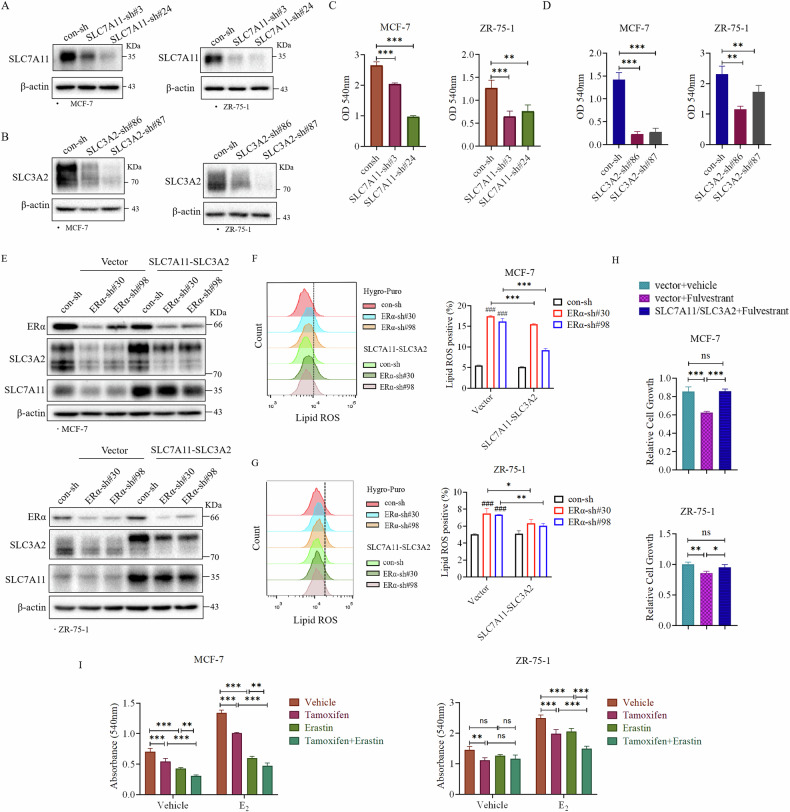
SLC7A11 and SLC3A2 mediate ERα inhibition of ferroptosis in ER+ breast cancer cells. SLC7A11 (**A**) and SLC3A2 (**B**) were knocked down by two different shRNAs in MCF-7 and ZR-75-1 cell lines, respectively. Knockdown of SLC7A11 (**C**) or SLC3A2 (**D**) inhibited the growth of ER+ breast cancer cells. MCF-7 and ZR-75-1 cells with SLC7A11 or SLC3A2 knockdown, respectively, were subjected to colony formation assay. **E** Overexpression of SLC7A11 and SLC3A2 in MCF-7 and ZR-75-1 cells with ERα knockdown. **F**, **G** Overexpression of SLC7A11 and SLC3A2 decreased the increase of Lipid ROS level caused by ERα knockdown. Lipid ROS levels in indicated cells in Fig. 5E were detected by flow cytometry. **H** Overexpression of SLC7A11 and SLC3A2 rescued the cell growth inhibited by ERα antagonist Fulvestrant. MCF-7 and ZR-75-1 cells with SLC7A11/SLC3A2 overexpression were treated with 1 µM Fulvestrant for 72 h. **I** Ferroptosis inducer Erastin enhanced the sensitivity of ER+ breast cancer cells to Tamoxifen treatment. MCF-7 and ZR-75-1 cells subjected to colony formation assay were treated with 10 nM E_2_ in presence of with vehicle, 5 μM Tamoxifen, 10 μM Erastin or in combination for 72 h. Data are shown as Mean ± SEM (*n* = 3). *P < 0.05, **P < 0.01, ***P < 0.001, ^###^*P* < 0.001; ns not significant.

To investigate whether SLC7A11 and SLC3A2 are involved in ERα-regulated ferroptosis, we examine the effects of knocking down SLC7A11 and SLC3A2 using two different shRNAs in ER+ breast cancer cells (Fig. 5A, B). Knockdown of SLC7A11 or SLC3A2 caused in a significant reduction in cell growth in MCF-7 and ZR-75-1 cells (Fig. 5C, D). Given that ERα depletion led to a pronounced induction of ferroptosis, we sought to determine whether overexpression of SLC7A11 and SLC3A2 could mitigate this effect. We co-overexpressed exogenous SLC7A11 and SLC3A2 in MCF-7 and ZR-75-1 cells with con-sh or ERα knockdown (Fig. 5E). The overexpression of the exogenous SLC7A11 and SLC3A2 not only resulted in a partial reduction of Lipid ROS levels induced by ERα knockdown (Fig. 5F, G) but also restored cell growth inhibited by the ERα antagonist Fulvestrant (Fig. 5H) and Tamoxifen (Fig. S2A). These findings supported the notion that ERα depletion induces ferroptosis and inhibits the growth of ER+ breast cancer cells in part through downregulation of SLC7A11 and SLC3A2. Lastly, we examined the effects of the Tamoxifen, Fulvestrant, and x_c_^−^ targeting drug Erastin on the growth of ER+ breast cancer cells. Compared with single Tamoxifen, or Fulvestrant, or Erastin treatment, the combination of the two drugs further inhibited the growth of ER+ breast cancer cells (Fig. 5I, S2B), indicating that inducing ferroptosis by targeting x_c_^−^ can increase the sensitivity of ER+ breast cancer cells in response to endocrine therapy.

### Expression of SLC7A11 and SLC3A2 were upregulated in endocrine resistant ER+ breast cancer

**Fig. 6:**
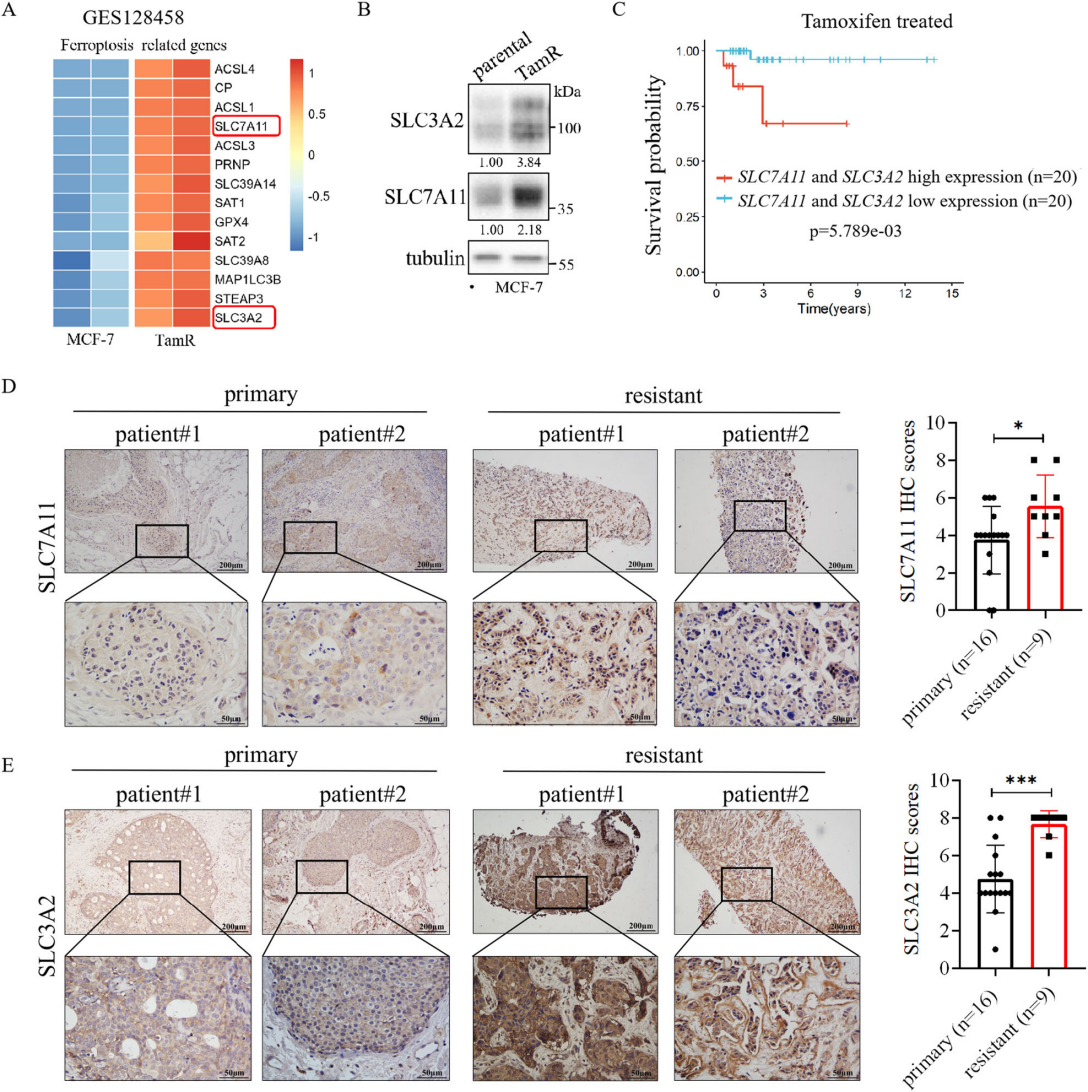
SLC7A11 and SLC3A2 are upregulated in endocrine resistant breast cancer. **A** Analysis of the GEO database (GSE128458) showed the expression of many ferroptosis-related genes including *SLC7A11* and *SLC3A2* are significantly elevated in Tamoxifen-resistant MCF-7 cells compared to the parental MCF-7 cells. **B** Western blot analysis showed that SLC7A11 and SLC3A2 protein levels are significantly higher in Tamoxifen-resistant than in the parental MCF-7 cell line. **C** Kaplan–Meier analysis of the survival time of breast cancer treated with Tamoxifen in the TCGA database with high or low mRNA levels of both *SLC7A11* and *SLC3A2*. **D**, **E** Immunohistochemistry analysis showed that SLC7A11 (**D**) and SLC3A2 (**E**) protein levels were significantly higher in endocrine therapy-resistant breast tumors compared with primary breast tumors. Scale bars, 200 μm, and 50 μm. SLC7A11 and SLC3A2 IHC staining were scored in breast tumor tissues from 25 patients. *P < 0.05, ***P < 0.001.

To investigate whether x_c_^−^ system is involved in resistance to endocrine therapy, we examined the relationship between the levels of SLC7A11 and SLC3A2, and endocrine resistance. Initial analysis of the GEO dataset revealed elevated expression of several ferroptosis-related genes including SLC7A11 and SLC3A2 in Tamoxifen-resistant MCF-7 cells compared to the parental MCF-7 cells (Fig. 6A, S3). In addition, the proteins levels of SLC7A11 and SLC3A2 were also significantly enhanced in the Tamoxifen-resistant (TamR) MCF-7-cells that we generated in the laboratory (Fig. 6B). Importantly, higher level of both of *SLC7A11* and *SLC3A2* mRNA was associated with poor survival in patients underwent Tamoxifen treatment (Fig. 6C). Furthermore, upregulation of SLC7A11 (Fig. 6D) and SLC3A2 (Fig. 6E) proteins was observed in endocrine-resistant breast tumors compared with primary ER+ breast tumors. These results suggest that the elevated levels of SLC7A11 and SLC3A2 in endocrine-resistant breast tumors may contribute to endocrine resistance.

### Ferroptosis inducer inhibited the growth of Tamoxifen resistant ER+ breast tumors

**Fig. 7:**
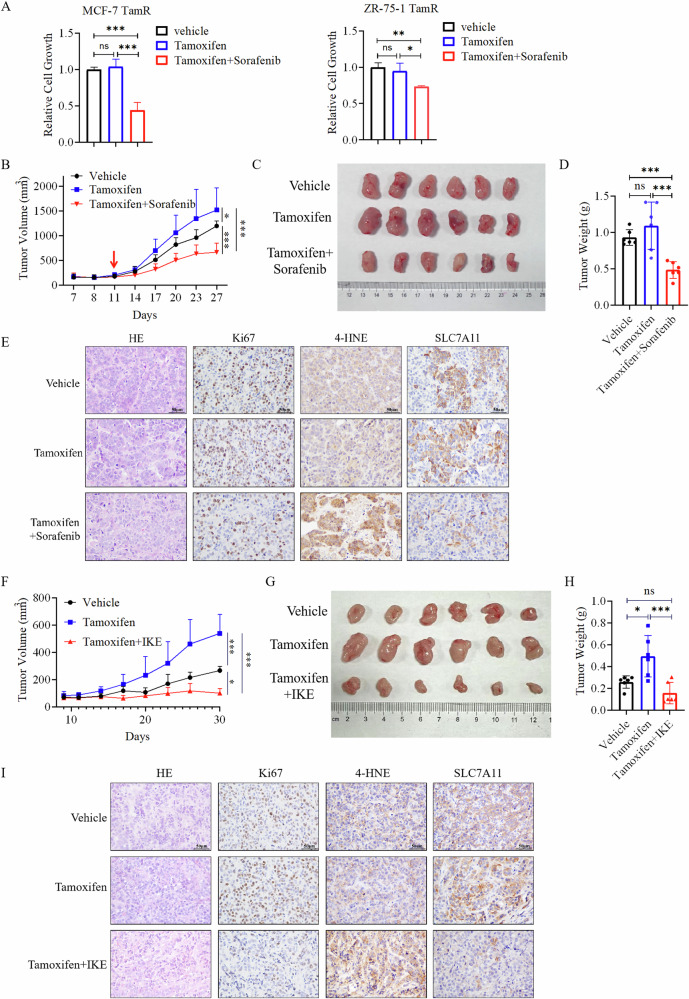
Ferroptosis inducer inhibits the growth of Tamoxifen resistant ER+ breast tumors. **A** Sorafenib inhibited the growth of Tamoxifen resistant (TamR) cells in vitro. Tamoxifen-resistant MCF-7 and ZR-75-1 cells were treated with 5 µM Tamoxifen and 5 µM Sorafenib for 72 h before being analyzed by the CCK8 reagent. Data are shown as Mean ± SEM (*n* = 3). **B**–**E** Sorafenib inhibited the growth of Tamoxifen-resistant cells in vivo. **B** The growth of the ZR-75-1-TamR tumors in response to treatment with Tamoxifen, or a combination of Tamoxifen and Sorafenib over the course of the experiment. The volumes of the tumors were recorded every 3 days, and the tumor growth curves were plotted. Tumors were dissected out from the euthanized mice at the end of the experiment, photographed (**C**), and weighted (**D**). **E** Representative images of Hematoxylin and Eosin (H&E) staining and immunostaining for Ki67, 4-HNE, and SLC7A11 in ZR-75-1-TamR tumors with the indicated treatments. Scale bar, 50 μm. **F**–**I** IKE inhibited the growth of Tamoxifen-resistant breast cancer cells in vivo. **F** The growth of the ZR-75-1-TamR tumors in response to treatment with Tamoxifen, or a combination of Tamoxifen and IKE over the course of the experiment. The volumes of the tumors were recorded every 3 days, and the tumor growth curves were plotted. Tumors were dissected out from the euthanized mice at the end of the experiment, photographed (**G**), and weighted (**H**). **I** Representative images of H&E staining and immunostaining for Ki67, 4-HNE, and SLC7A11 in ZR-75-1-TamR tumors with the indicated treatments. Scale bar, 50 μm. *P < 0.05, **P < 0.01, ***P < 0.001; ns not significant.

Since overexpression of SLC7A11 and SLC3A2 also reduced sensitivity of ER+ breast cancer cells to Fulvestrant treatment (Fig. 5H), we hypothesized that drug targeting SLC7A11 and SLC3A2 should have efficacy against endocrine-resistant breast tumors by inducing ferroptosis. Despite its widespread use in cell culture studies, SLC7A11 inhibitor Erastin cannot be used for animal studies due to its poor metabolic stability and low solubility in vivo [13]. Therefore, we assessed the effect of Sorafenib, another SLC7A11-targeting ferroptosis inducer that has been used for liver cancer therapy in clinic, on Tamoxifen resistant ZR-75-1 cells in vitro and in vivo. Sorafenib treatment significantly inhibited the growth of Tamoxifen resistant MCF-7 and ZR-75-1 cells in vitro (Fig. 7A), treatment with Sorafenib alone also inhibited cell growth (Fig. S4). However, total tyrosyl phosphorylation (Figure S5A) and ERK phosphorylation (Figure S5B) were not altered in Tamoxifen resistant cell with Sorafenib treatment, suggesting that the effect of Sorafenib on cell growth is unlikely through inhibition of RTKs or RAF. Subsequently, the efficacy of Sorafenib was evaluated in the Tamoxifen resistant ZR-75-1-TamR xenograft model (Fig. S6A). While Tamoxifen did not decrease the growth of ZR-75-1-TamR tumors, the combined administration of Sorafenib with Tamoxifen significantly inhibited the tumor growth compared to Tamoxifen treatment alone (Fig. 7B–D). Immunohistochemistry analysis revealed that Sorafenib treatment significantly reduced the levels of SLC7A11 and Ki67, a proliferation marker, and increased the level of 4-HNE, a marker of ferroptosis, in tumor tissues (Fig. 7E).

To further support the efficacy of system x_c_^−^ inhibitor on Tamoxifen tumors, IKE, a specific system x_c_^−^ inhibitor with excellent metabolic stability was used in the ZR-75-1-TamR xenografts (Fig. S6B). Compared to Tamoxifen alone, IKE adminstration in combination with Tamoxifen significantly inhibited cell growth (Fig. 7F–H). Immunohistochemistry analysis revealed that IKE treatment resulted in significant reductions in SLC7A11 and Ki67 levels, as well as increases in 4-HNE levels in tumor tissues (Fig. 7I). These data indicate that x_c_^−^ targeting ferroptosis inducing drug has efficacy against endocrine resistant ER+ breast cancer.

## Discussion

Breast cancer is a common form of cancer globally, with approximately 70% of cases being classified as ER+ breast cancer that depends on estrogen for growth. However, the primary challenge faced by ER+ breast cancer patients is the development of endocrine resistant. Our research revealed that estrogen/ERα signaling inhibits ferroptosis in ER+ breast cancer by upregulating the expression of SLC7A11 and SLC3A2. Endocrine resistant ER+ breast cancer has elevated levels of SLC7A11 and SLC3A2. Ferroptosis inducer targeting SLC7A11 can inhibit the growth of endocrine resistant ER+ breast tumors.

Recent studies have indicated that ferroptosis, a form of regulated cell death, plays a significant role in the progression and management of various cancers, including breast cancer. The combination of a BET inhibitor (+)-JQ-1 and a proteasome inhibitor bortezomib (BTZ) has been shown to decrease GPX4 expression, thereby inducing ferroptosis in TNBC [18]. Data in the literature suggests that TNBC is more susceptible to ferroptosis compared to ER+ breast cancer. Approximately 40-50% of ER+ breast cancers harbor *PI3KCA* mutations, which are associated with increased GSH synthesis and resistance to oxidative stress, ultimately reducing the sensitivity of ER+ breast cancer to ferroptosis [21]. Our study showed that estrogen stimulation enhanced cystine uptake and increased the GSH/GSSG ratio (Figs. 1I, 1J), which may be one reason why ER+ breast cancer cells exhibited higher levels of GSH compared to TNBC cells (Fig. 1A). The system x_c_^−^, consisting of the proteins SLC7A11 and SLC3A2, facilitates the exchange of transfer between cystine and glutamine, thereby playing a crucial role in cystine uptake and promoting GSH synthesis. Analysis of the dataset in Gene Expression Omnibus (GEO) showed that *SLC7A11* and *SLC3A2* are two ferroptosis-related genes regulated by estrogen (Fig. 3A). Since ERα transcriptionally upregulated SLC7A11 and SLC3A2 expression (Fig. 4), we believe that estrogen/ERα inhibits ferroptosis mainly through the x_c_^−^-GPX4 axis in ER+ breast cancer. However, ERα knockdown resulted in the elevation of Fe^2+^ level in cells (Fig. 1F), suggesting that ERα signaling also regulates the homeostasis of cellular iron in the context of ferroptosis. Consistent with this notion, it has been reported that ERα can negatively regulate the level of transferrin receptor TFRC, enhancing the resistance of ER+ breast cancer cells to ferroptosis [22]. Since the combination of overexpressing SLC7A11 and SLC3A2 partially alleviated ferroptosis induced by ERα knockdown (Figs. 5F, 5G), it is also possible that estrogen/ ERα regulates ferroptosis through x_c_^−^-GPX4 independent pathway in ER+ breast cancer cells. Consistent with this notion, a recent study reported that membrane-bound O-acyltransferase domain containing1 and 2 (MBOAT1/2), which is regulated by ERα, inhibits ferroptosis through phospholipid remodeling independently of GPX4 [23]. However, we did not observed that knockdown of ERα inhibited the expression of MBOAT1 or 2 in MCF-7 cells (data not shown).

**Fig. 8:**
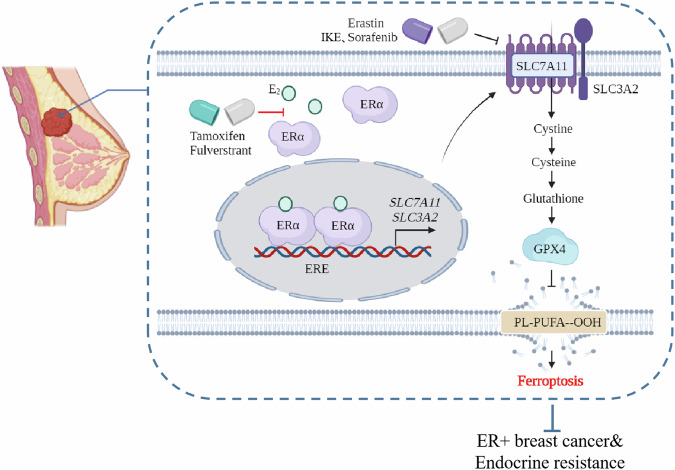
Working model. Estrogen inhibits ferroptosis via transcriptionally upregulation of the system x_c_^−^ (SLC7A11 and SLC3A2) in ER+ breast cancer cells. Targeting SLC7A11 and SLC3A2 may offer a novel therapeutic option for patients with ER+ breast cancer, particularly those with endocrine resistance.

Published studies reveal that distinct mechanisms involved in regulation of SLC7A11 and SLC3A2 expression are context dependent. Wang et al. demonstrated that the E3 ubiquitin-protein ligase TRIM3 functions as a tumor suppressor by degrading SLC7A11, thereby inhibiting the tumorigenesis of non-small cell lung cancer (NSCLC) [24]. The cell polarity protein SCRIB regulates the intracellular localization of SLC3A2 in ER+ breast cancer cells, without affecting its expression level [25]. The glycosyltransferase B3GNT3 facilitates the glycosylation of SLC3A2, leading to the stabilization of the SLC3A2 protein and increased interaction between SLC3A2 and SLC7A11 [26]. In addition, multiple transcription factors, such as ATF3, ATF4, P53, Nrf2, and c-Myc, have been identified as the regulators of SLC7A11 and SLC3A2 transcription [11, 27–30]. Interestingly, a recent study showed that CDK4/6 inhibitors blocked SLC7A11 expression by inhibiting SP1 binding to the SLC7A11 promoter and induced ferroptosis in luminal A breast cancer cells [31]. ERα is the key driver of ER+ breast cancer. Our study demonstrated that ERα binds to the ERE present in the promoter regions and enhances the transcription of *SLC7A11* and *SLC3A2* (Fig. 4), inhibiting ferroptosis and contributing to the growth of ER+ breast cancer cells. We uncover a novel mechanism by which estrogen regulates ferroptosis in ER+ breast cancer (Fig. 8).

ER+ breast cancer patients may develop endocrine resistance due to different mechanisms. A published report has shown that the NF-κB member, RelB, plays a role in upregulating GPX4 expression and suppressing ferroptosis, thereby increasing the resistance of ER+ breast cancer cells to Tamoxifen [32]. The expression of the cell polarity protein SCRIB can mediate endocrine resistance in ER+ breast cancer cells. SCRIB upregulated by estrogen signaling interacts with SLC3A2 to modulate leucine amino acid transport, contributing to the proliferation and tamoxifen resistance of ER+ breast cancer cells [25]. Our study revealed that SLC7A11 and SLC3A2 were found to be upregulated in endocrine resistant breast tumors and cancer cells (Fig. 6). In addition to these genes, other ferroptosis-related genes were also upregulated in Tamoxifen resistant MCF-7 cells (Fig. 6A), supporting that altered ferroptosis plays a role in endocrine resistance. Further investigation into the role of other ferroptosis-related proteins in endocrine resistance is likely warranted.

Sorafenib, an FDA-approved multi-target kinase inhibitor, is the first-line systemic therapy for advanced HCC. It inhibits the transport activity of SLC7A11, induces ferroptosis, and suppresses tumor growth [33]. Although Sorafenib is a kinase inhibitor with multiple targets including RTKs and RAF, its treatment did not affect the total tyrosyl phosphorylation and ERK phosphorylation in TamR cells (Figure S5), indicating that inhibition of TamR ER+ breast cancer cell growth by Sorafenib under the concentration used is not through inhibition of RTKs and RAF. Our findings indicate that Sorafenib can effectively inhibit the growth of ZR-75-1-TamR cells both in vitro and in vivo. Furthermore, combination of IKE and Tamoxifen dramatically inhibit the growth of ZR-75-1-TamR cells in vitro (Fig. 7). It is interesting to note the RESILIENCE trial using sorafenib with Capecitabine did not improve PFS and OS for patients with advanced HER2 negative breast cancer, in which ~69% of the patients were with HR positive breast cancer [34]. Based on our data, we believe that the RESILIENCE trial would have achieved better outcome for the patients if it were designed to treat HR+ patients with Sorafenib together with hormonal therapy versus Placebo with hormonal therapy. Our study implicates that targeting the estrogen-dependent system x_c_^−^, specifically SLC7A11 and SLC3A2, with IKE through induction of ferroptosis in ER+ breast cancer cells, should offer a new therapeutic option for patients with ER+ breast cancer, particularly those with endocrine resistance.

## Materials and methods

### Cell lines

MCF-7, ZR-75-1, and T47D human breast cancer cell lines and HEK293T17 cells were purchased from the American Type Culture Collection (ATCC, Maryland, USA). Tamoxifen-resistant MCF-7 and ZR-75-1 cells were generated as described below. Parental MCF-7 were cultured in growth medium containing 5% fetal bovine serum (FBS) with increasing amount of Tamoxifen, starting from 0.5 μM initially, finally up to 5 μM in a period of around 6 months. Parental ZR-75-1 cells were cultured in Phenol-red free medium containing 10% Dextran-coated charcoal-stripped FBS (DCC) with increasing concentration of Tamoxifen, starting from 0.5 μM initially, and finally up to 5 μM in a period of about 6 months. The parental MCF-7 cells and HEK293T17 cells were cultured in DMEM (Gibco, California, USA) with 5% FBS (Excell, Suzhou, China) supplemented with 100 units/mL penicillin and 100 µg/mL streptomycin (Beyotime Biotechnology, Jiangsu, China). ZR-75-1 and T47D cells were cultured in DMEM with 10% FBS supplemented with 100 units/mL penicillin and 100 µg/mL streptomycin. Tamoxifen-resistant MCF-7 cells were maintained in DMEM medium with 5% FBS, 100 units/mL penicillin, 100 µg/mL streptomycin, and 5 μM Tamoxifen (Selleck, Houston, USA). Tamoxifen-resistant ZR-75-1 cells were cultured in phenol-red free DMEM (Sigma, Darmstadt, Germany) supplemented with 10% DCC, 100 units/mL penicillin, 100 µg/mL streptomycin and 5 μM Tamoxifen. Cells were incubated at 37 °C in a 7.5% CO_2_ atmosphere. All cell lines were characterized by DNA fingerprinting and isozyme detection.

### Plasmids

The pGL3-basic luciferase reporter plasmid (pGL3-luc) was described in our previous research [35]. DNA fragments of *SLC7A11* (-1498 to -123) and *SLC3A2* (-1407 to -120) promoter regions were amplified from MDA-MB-231 genomic DNA by PCR and subcloned into the pGL3-luc plasmid. DNA fragments of ERα-shRNA#30, ERα-shRNA#98, SLC7A11-shRNA#3, SLC7A11-shRNA#24, SLC3A2-shRNA#86 and SLC3A2-shRNA#87 (sequences from Sigma-Aldrich website) were subcloned into the *Eco*R I and *Age* I digested pLKO.1 vector, respectively. The cDNA for *SLC7A11* coding region was amplified from ZR-75-1 breast cancer cell line using standard RT-PCR and ligated into *EcoR* I-*Sal* I linearized retroviral pBabe-hygro vector, generating the pBabe-hygro-SLC7A11. The cDNA for SLC3A2 coding region was amplified from ZR-75-1 breast cancer cell line using standard RT-PCR and ligated into *BamH* I-*EcoR* I linearized pBabe-puro retroviral vector, generating the pBabe-puro-SLC3A2. All primer sequences for PCR amplification and cloning in this study were listed in the Supplementary information. All of the generated plasmids were verified by DNA sequencing (GENEWIZ, Jiangsu, China).

### Antibodies and reagents

The anti-β-actin (cat#3700), anti-α-tubulin (cat#3873), anti-SLC7A11 (cat#12691), anti-ERα (cat#13258), phospho-Tyr-100 (cat#9411), and phospho-ERK (Thr202/Tyr204) (cat#4370) were purchased from Cell Signaling Technology (Danvers, MA, USA). Anti-SLC3A2 (cat#15193) antibodies were from Proteintech (Wuhan, China). Ferrostatin-1, Sorafenib, Erastin, and Imidazole ketone erastin (IKE) (HY-114481) were purchased from MedChemExpress (N.J, USA). Tamoxifen and Fulvestrant were purchased from Selleck.

### Retrovirus/lentivirus production and viral infection

The production of retroviruses and lentiviruses were performed as described [36, 37]. ER+ breast cancer cells were incubated with retrovirus or lentiviral supernatants in the presence of 8 μg/mL of polybrene (Sigma) for 16 h. Twenty-six hours post-infection, stable pools of cells were selected with 1.2 μg/mL puromycin (Invitrogen, San Diego, USA) for MCF-7 cells and 1.4 μg/mL puromycin for ZR-75-1 cells for 3 days or 100 μg/mL hygromycin for MCF-7 and ZR-75-1 cells for 7 days before being used for subsequent experiments.

### Cell growth and cell colony formation assay

Cell growth was assessed by using the Cell Counting Kit-8 (CCK-8) (Dojindo Laboratories, Kumamoto, Japan) according to the manufacturer’s instructions. Cells were seeded at a density of 5×10^3^/well in 200 μL of medium into 96-well plates (BIOFIL, Guangzhou, China), and treated with Erastin or ferrostatin-1 for the indicated time. Subsequently, CCK-8 reagent (20 μL) was added to each well, incubated for 2 h, and analyzed at 450 nm using Varioskan flash microplate reader. Relative cell growth was presented as the absorbance. For colony formation assay, cells were cultured in 24-well tissue culture plates for a week to form colonies before being fixed with 10% neutral formalin, stained with 0.5% crystal violet solution, and having the dye extracted by adding 10% acetic acid. The absorbance at 540 nm was measured using a Varioskan flash microplate reader.

### Quantitative real-time PCR (q-PCR) for mRNA level

Total RNAs were extracted from MCF-7 and ZR-75-1 cells using RNA-easy Isolation Reagent (Vazyme, Nanjing, China) according to the manufacturer’s instructions. cDNA was synthesized from 1 μg of purified RNA using HiScript II Q RT SuperMix (Vazyme) and subjected to quantitative real-time PCR (q-PCR) for analyzing the relative mRNA levels of specific genes with proper primers using ChamQ Universal SYBR qPCR Master Mix (Vazyme). Q-PCR data are uploaded as the “original data”.

### Western blot

Cells were harvested and lysed by 1× SDS sample buffer. Equal amounts of cell protein lysates were separated by SDS PAGE and transferred to PVDF membranes (Millipore, Darmstadt, Germany). The membranes were blocked in 5% skim milk for 1.5 h at room temperature and then immunoblotted with the appropriate primary antibodies at 4°C overnight. After three washes with TBST, membranes were incubated with horseradish peroxidase-conjugated secondary antibodies (Jackson Immunoresearch, Pennsylvania, USA) for 1.5 h at room temperature. After three washes with TBST, membranes were detected by Enhanced Chemiluminescence with images captured by the ChemiDoc MP imaging system (Bio-rad, California, USA). Full and uncropped western blots are uploaded as the “original data”.

### Measurement of lipid peroxidation

MCF-7 and ZR-75-1 cells were seeded in 12-well plates at an appropriate density and subjected to indicated treatments followed by staining with 10 μM BODIPY C11 (Thermo Fisher #D3861, Massachusetts, USA) for 30 min. Cells were harvested with trypsin digestion, pelleted, and washed with PBS before being analyzed by flow cytometry (BD Accuri C6). Oxidation of BODIPY C11 resulted in a shift of the fluorescence emission peak from 590 nm to 510 nm.

### Measurement of iron concentration

The intracellular iron concentration was quantified using a Total Iron Content Colorimetric Assay Kit (Applygen #E1042, Beijing, China) according to the manufacturer’s instructions.

### Transmission electron microscopy

Cells cultured in a 10-cm plate were harvested with trypsin digestion, and fixed with 2.5% glutaraldehyde at 4 °C for more than 4 h. Finally, ultrathin sections were cut, and the morphological changes of mitochondria were examined under the TEM (HT7800, Hitachi, Japan).

### Measurement of cystine uptake

Cells were plated in a black 96-well plate to prevent light from leaking into the adjacent wells during measurement. Cystine uptake by cells was measured using the Cystine uptake assay kit (Dojindo Laboratories) according to the manufacturer’s instructions. The fluorescence was measured using a fluorescence microplate reader.

### Measurement of intracellular glutathione (GSH) content

Total cellular glutathione levels were detected using the GSSG/GSH Quantification Kit (Dojindo, Japan) according to the manufacturer’s instructions.

### Luciferase reporter assay

MCF-7 cells were seeded into 24-well plates in triplicate with phenol-red free DMEM supplemented with 5% DCC. Cells were cotransfected with pGL3-*SLC7A11* promoter-luc or pGL3-*SLC3A2* promoter-luc together with TK-renilla luciferase (internal control) plasmids (pGL3-luc plasmid: TK-renilla plasmid = 10:1). Sixteen hours post-transfection, cells were replenished with fresh medium containing 10 nM estrogen (E_2_). After 24 h, cells were lysed and subjected to luciferase activity using a Luc-Pair ™ Duo-Luciferase HS Assay Kit (GeneCopoeia, MD, USA) according to the manufacturer’s instructions.

### Chromatin Immunoprecipitation (ChIP) assay

ChIP assay was performed as previously described [38]. ChIP DNA was isolated and subjected to q-PCR analysis using primers designed to amplify the promoter regions of *SLC7A11* and *SLC3A2* containing the predicted estrogen responsive elements. Relative occupancy values were calculated by comparing the levels of immunoprecipitated DNA to the input DNA. In ChIP experiments involving anti-ERα (sc-8005, Santu Cruz Biotechnology, Texas, USA), the values were normalized to those obtained with control IgG.

### Hematoxylin-eosin (H&E) and Immunohistochemistry (IHC)

For H&E staining, tissue sections were immersed in hematoxylin for 1 min, washed with water for 5 min, and stained with eosin for 20 seconds. The slides were mounted after dehydration with different concentrations of ethanol. Formalin-fixed and paraffin-embedded (FFPE) primary breast tumor samples and endocrine therapy resistant breast tumor samples were from the Pathology Department at The First Affiliated Hospital of Wenzhou Medical University and The Third Affiliated Hospital of Wenzhou Medical University. FFPE breast tumor sections (4 μm thickness) were subjected to immunohistochemistry (IHC) as described [38] with some modifications. Antigen retrieval was performed in 10 mM citrate buffer, pH 6.0 using pressure cooker (at 125 °C for 5 min). Rabbit anti-SLC7A11 polyclonal antibodies (cat#26864, Proteintech) were used at 1:50 dilution. Rabbit anti-SLC3A2 polyclonal antibodies (cat#15193, Proteintech) were used at 1:300 dilution. Rabbit anti-Ki67 monoclonal antibodies (D2H10, Cell Signaling Technology) were used at 1:800 dilution. Rabbit anti-4-HNE polyclonal antibodies (ab46545, Abcam, Cambridge, UK) were used at 1:100 dilution.

### Animal experiments

Six-week-old female BALB/c nude mice were purchased from Beijing Vital River Laboratories Animal Technology (Beijing, China). Tamoxifen-resistant ZR-75-1 cells (4 × 10^6^ cells) mixed with 1:1 Matrigel (Corning, cat#354234) were injected into the fourth mammary fat pads of mice. A 60-day release estrogen pellet (1.5 mg/pellet, Innovative Research of America, Sarasota, Florida, USA) was implanted onto the backs of mice 2 days before cell injection. For examining the efficacy of Sorafenib, after the sizes of the tumors reached around 200 mm^3^, mice were randomized into three groups (six mice per group). They were treated with vehicle, Tamoxifen (30 mg/kg, oral gavage), or a combination of Tamoxifen and Sorafenib (30 mg/kg, oral gavage). For examining the efficacy of IKE, after the size of the tumors reached around 100 mm^3^, mice were randomized into three groups (six mice per group): 1) Vehicle group with vehicle A (5% DMSO, 95% corn oil) (oral gavage) every three days and daily i.p. with vehicle B [65% D5W (5% dextrose in water), 5% Tween-80, 30% PEG-400], 2) Tamoxifen group with 30 mg/kg Tamoxifen dissolved in vehicle A (oral gavage), 3) Tamoxifen + IKE group with 30 mg/kg Tamoxifen (oral gavage) every three days and daily i.p. with 40 mg/kg IKE dissolved in vehicle B. Tumor volumes were measured every 3 days, and were calculated according to the formula volume = length × width^2^/2. The mice were euthanized at the end of the experiment, and xenografted tumors were dissected, weighed, and photographed. The animal study was approved by the Institutional Animal Care and Use Committee of Wenzhou Medical University.

### TCGA and GEO datasets

Transcriptome data of breast cancer (BRCA) cohort from TCGA (https://www.cancer.gov) and Gene Expression Omnibus (GEO) dataset (GSE173300, GSE128458, and GSE144378) (http://www.ncbi.nlm.nih.gov/geo) were analyzed. The survival time of breast cancer patients with different levels of SLC7A11 and SLC3A2 mRNA (GSE26304, GSE18229, GSE159956, GSE25055) was depicted by PanCanSurvPlot (https://smuonco.shinyapps.io/PanCanSurvPlot/).

GSE173300: MCF-7 cells treated with several combinations of steroids.

GSE128458: Parental- and TamR- ER+ breast cancer cells.

GSE144378: Parental- or TamR- ER+ breast cancer cells treated with or not treated with TKI sapitnib.

GSE26304: Breast cancer samples.

GSE18229: Claudin-low Intrinsic Subtype of Breast Cancer.

GSE159956: breast cancer samples from human patients.

GSE25055: HER2-negative breast cancer cases treated with taxane-anthracycline chemotherapy pre-operatively and endocrine therapy if ER-positive.

### Statistical analysis

Statistical analyses were performed using Prism, version 8.0. Student t-test was used to compare data between two groups. One-way ANOVA with Bonferroni’s multiple comparison test correction was used to analyze data among multiple groups. Two-way ANOVA was used to analyze differences with two independent factors. All statistical tests were two-sided, and *P* < 0.05 was considered statistically significant. The results shown are representative of three independent experiments.

## Supplementary information

supplementary figures

supplementary information

original data

## Data Availability

All data generated or analyzed during this study are included in this published article. Materials generated in this study will be freely available to any researcher upon reasonable request.
