# Supplementary material for: Targeting estrogen-regulated system xc− promotes ferroptosis and endocrine sensitivity of ER+ breast cancer
Source: Cell Death Dis. 2025 Jan 20;16(1):30. doi: 10.1038/s41419-025-07354-0 (PMC11756422; doi:10.1038/s41419-025-07354-0)
Supplement: Supplementary file 1 — supplementary figures [file 41419_2025_7354_MOESM1_ESM.doc]

**Supplementary figures**


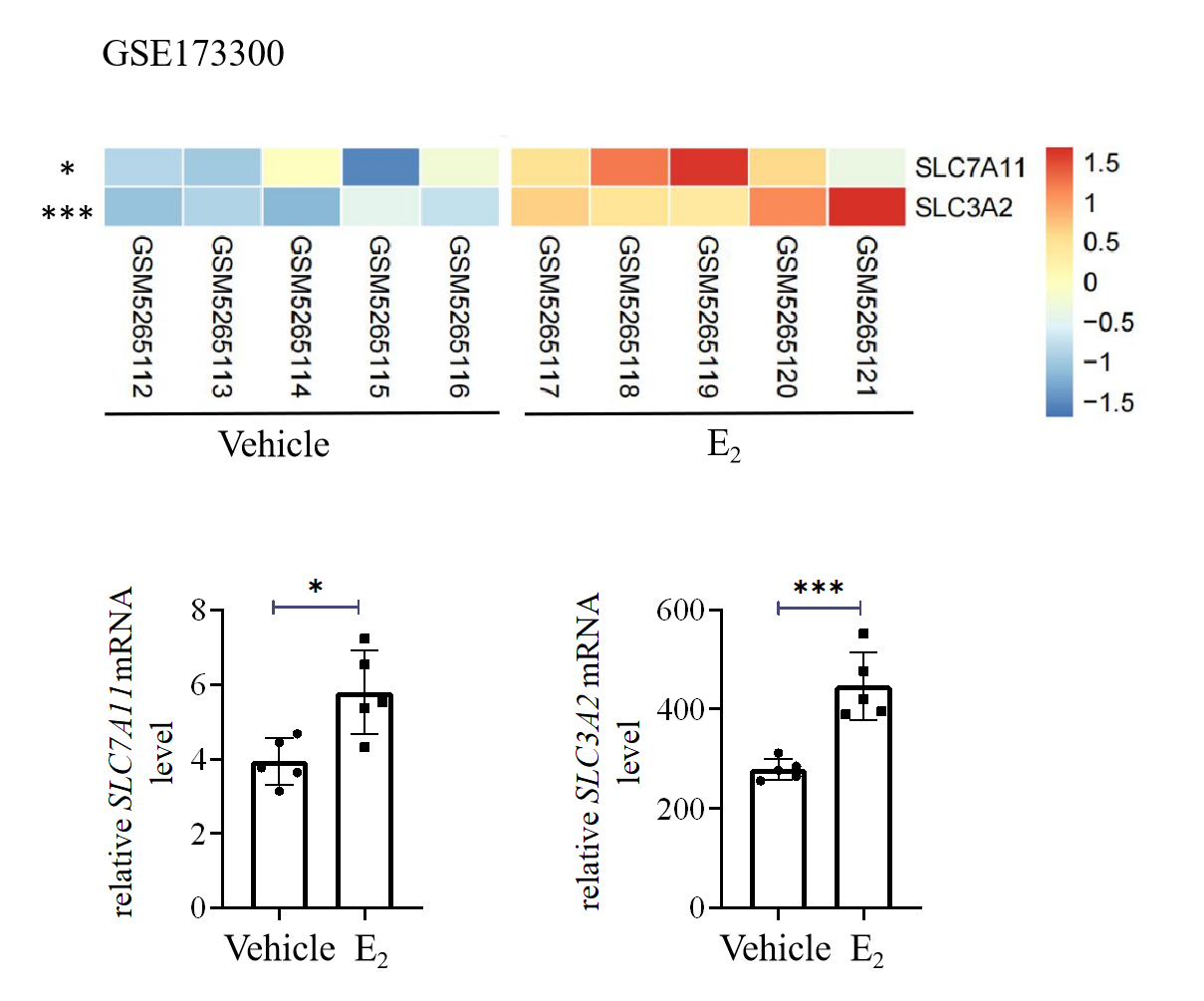


Figure S1. E_2_ treatment increases the mRNA levels of *SLC7A11* and *SLC3A2* in MCF-7 cells from the GSE173300 datasets. A heatmap of relative *SLC7A11* and *SLC3A2* expression is shown above, followed by statistical analysis of *SLC7A11* and *SLC3A2* levels. Data are shown as Mean ± SEM (n=5). *P<0.05, ***P<0.001.


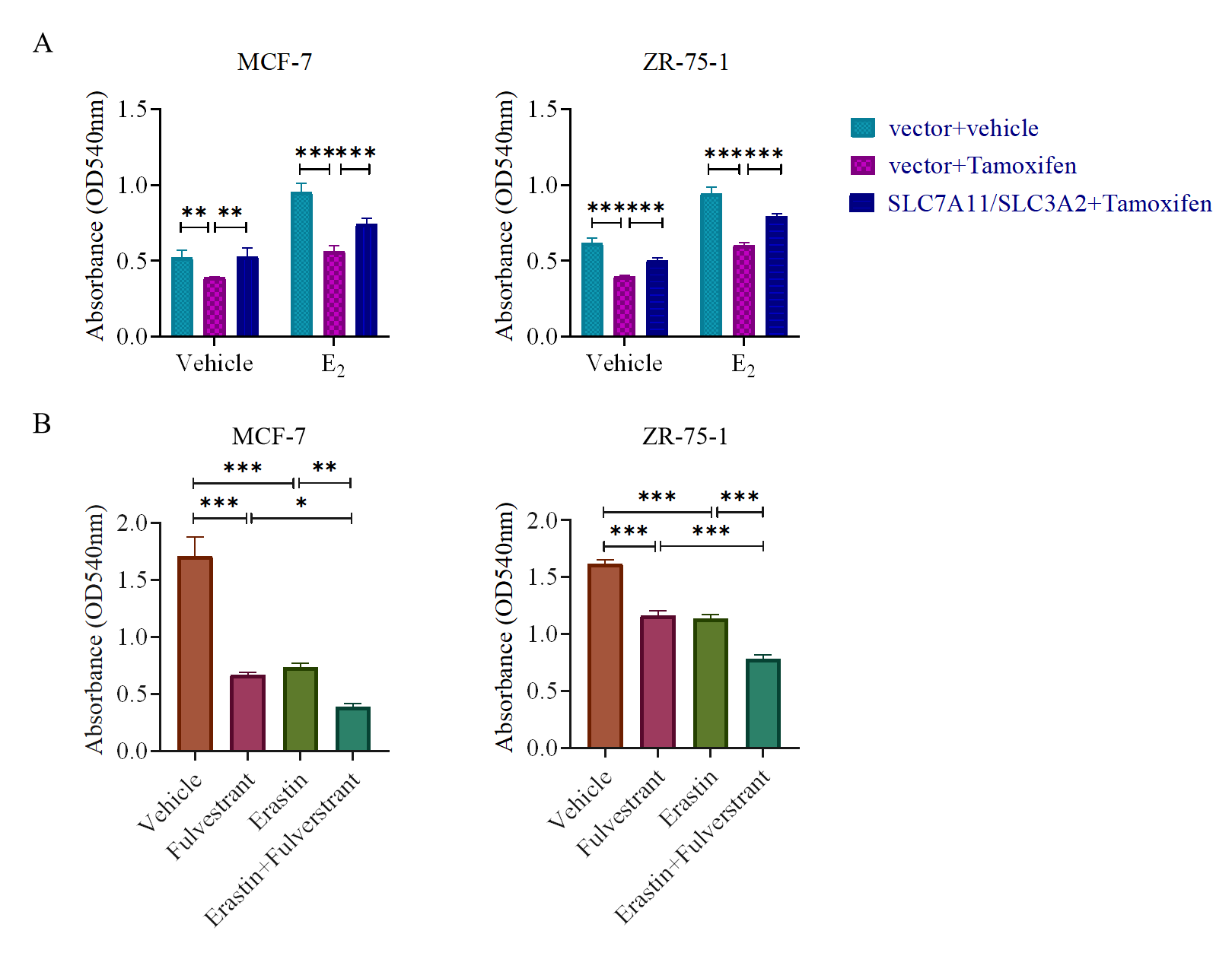
Figure S2. Xc^-^ system is important in regulating the sensitivity of ER+ breast cancer cells to endocrine therapy.

(A) Overexpression of SLC7A11 and SLC3A2 rescued the cell growth inhibited by Tamoxifen. MCF-7 and ZR-75-1 cells with SLC7A11/SLC3A2 overexpression subjected to colony formation assay were treated with 10 nM E_2_ in the presence of vehicle or 5 μM Tamoxifen for 72 h.

(B) Ferroptosis inducer Erastin enhanced the sensitivity of ER+ breast cancer cells to Fulvestrant. MCF-7 and ZR-75-1 cells subjected to colony formation assay were treated with vehicle, 1 μM Fulvestrant, 10 μM Erastin or in combination for 72 h.

Data are shown as Mean ± SEM (n=3). **P<0.01, ***P<0.001.


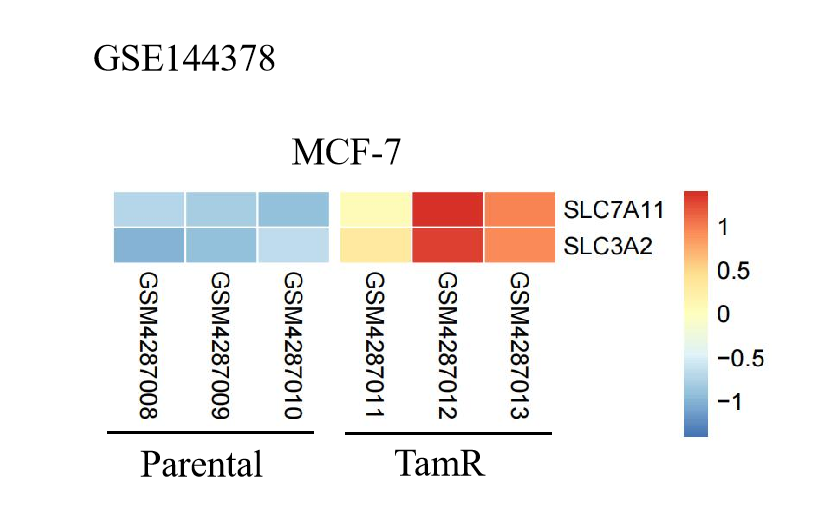


Figure S3. *SLC7A11* and *SLC3A2* mRNA levels are upregulated in TamR cells compared to parental cells.

The GEO (GSE144378) demonstrated that *SLC7A11* and *SLC3A2* mRNA levels were significantly elevated in TamR cells in comparison to the parental cells.


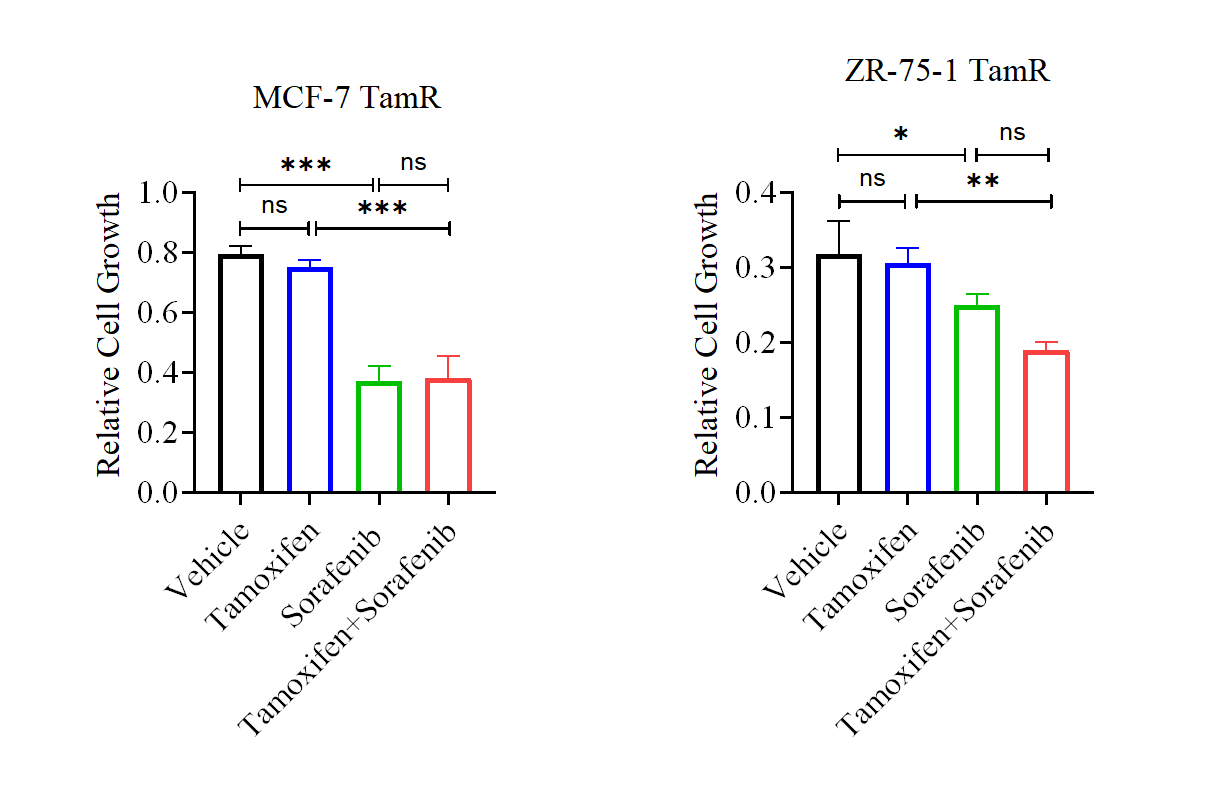


Figure S4. Sorafenib inhibits the growth of Tamoxifen resistant breast cancer cells *in vitro*.

Tamoxifen-resistant MCF-7 and ZR-75-1 cells were treated with 5 µM Tamoxifen or 5 µM Sorafenib alone or together for 72 h before being analyzed by the CCK8 reagent. Data are shown as Mean ± SEM (n=3). *P<0.05, **P<0.01, ***P<0.001, ns= no significant difference.


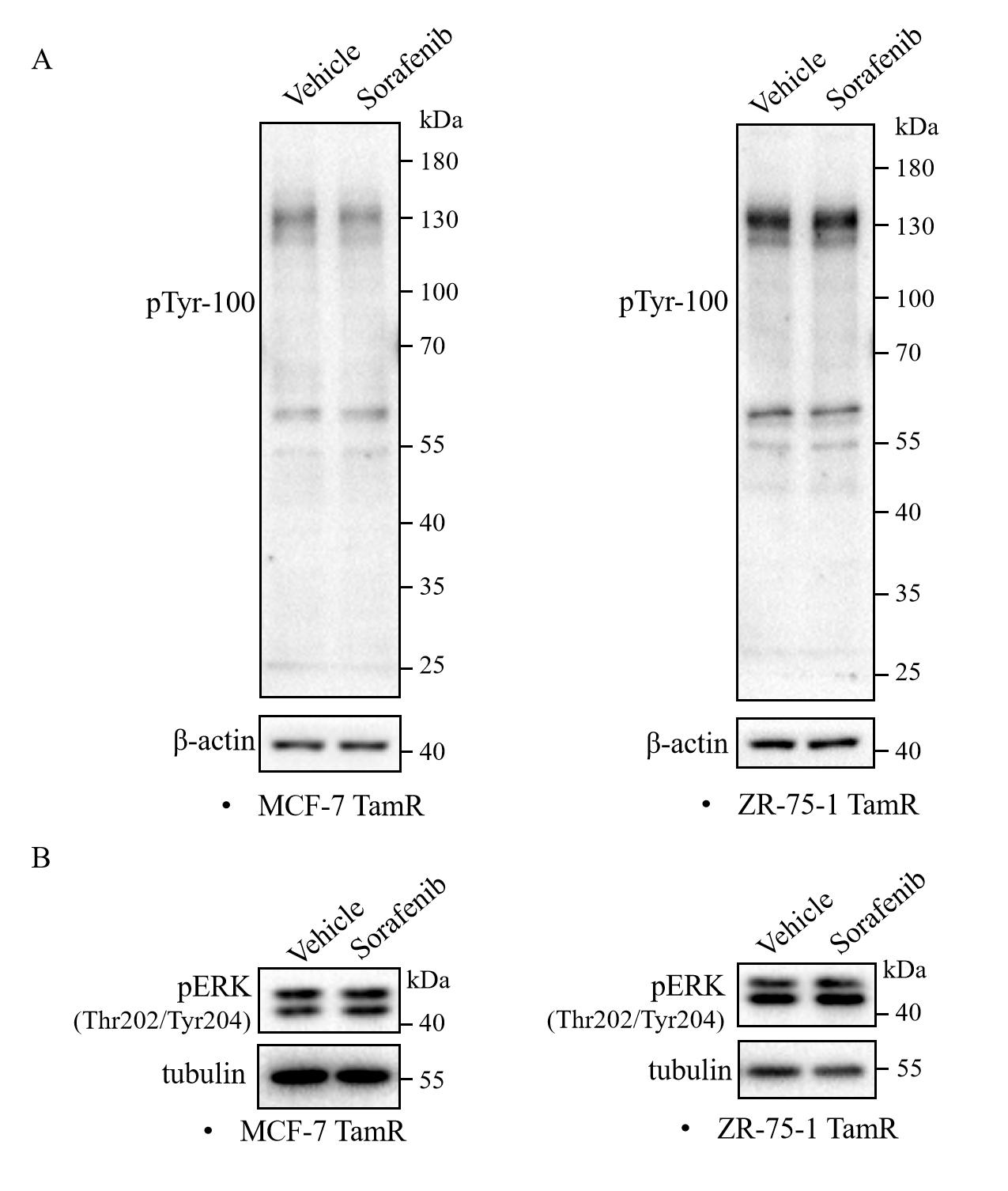


Figure S5. Sorafenib does not alter the total cellular tyrosyl phosphorylation and ERK phosphorylation in Tamoxifen resistant cells.

(A) MCF-7-TamR and ZR-75-1-TamR cells were treated with 5 µM Sorafenib for 24 h, and then were subjected to western blot using antibodies against tyrosyl phosphoryalted protein (pTyr-100) and β-actin.

(B) The same cells as (A) were subjected to western blot using antibodies against phosphorylated ERK, pERK (Thr202/Tyr204) and α-tubulin.


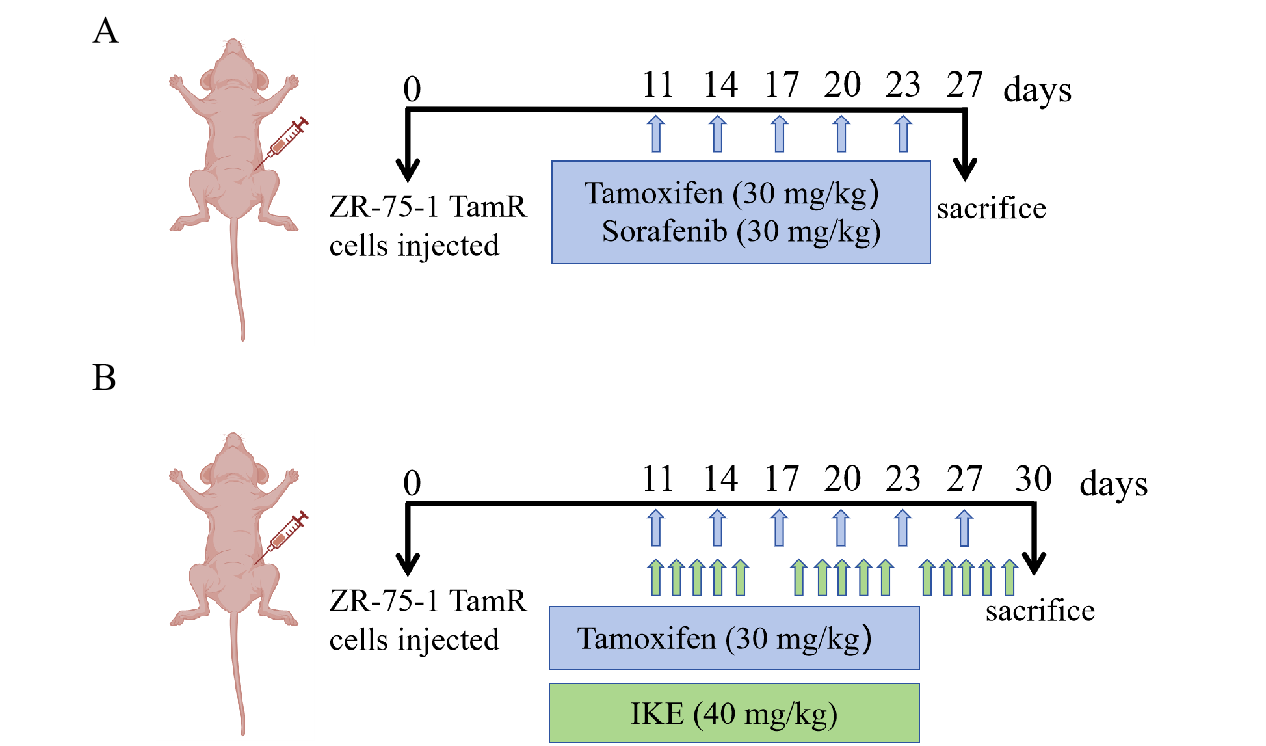


Figure S6. Drug treatment plans for the ZR-75-1 TamR tumor model established in BABL/C nude female mice.

(A) ZR-75-1 TamR tumor model established were treated with Vehicle, Tamoxifen, or a combination of Tamoxifen and Sorafenib at the indicated time points.

(B) ZR-75-1 TamR tumor model established were treated with Vehicle, Tamoxifen, or a combination of Tamoxifen and IKE at the indicated time points.
