# Supplementary material for: Targeting estrogen-regulated system xc− promotes ferroptosis and endocrine sensitivity of ER+ breast cancer
Source: Cell Death Dis. 2025 Jan 20;16(1):30. doi: 10.1038/s41419-025-07354-0 (PMC11756422; doi:10.1038/s41419-025-07354-0)
Supplement: Supplementary file 2 — supplementary information [file 41419_2025_7354_MOESM2_ESM.docx]

**Supplementary Information**

**The primer pairs were used for PCR cloning of *SLC7A11* or *SLC3A2* promoter:**

**pSLC7A11- promoter 1.5kb**

Forward: 5’ -CGACGCGTAGCACACCCTTACAAGAACTG-3’

Reverse: 5’ -GAAGATCTAACTCTCTACCCACAACTCCC-3’

**pSLC3A2-promoter 1.5kb**

Forward: 5’ -CGACGCGTGGATGGCAAAGCATTGAGGTG-3’

Reverse: 5’ -GAAGATCTCTCAGCTTCCTCATGGGCTTG-3’

**shRNA sequence information:** **(sequences from sigma)**

ERα-shRNA#30: 5’-CTACAGGCCAAATTCAGATAA-3’

ERα-shRNA#98: 5’-GCCCTACTACCTGGAGAACGA-3’

SLC7A11-shRNA#3: 5’ -CCCTGGAGTTATGCAGCTAAT-3’

SLC7A11-shRNA#24: 5’-GCACCCTTTGACAATGATAAT-3’

SLC3A2-shRNA#86: 5’-GCTGGGTCCAATTCACAAGAA-3’

SLC3A2-shRNA#87: 5’-CTAGCTCATACCTGTCTGATT-3’

**The primer pairs were used for detecting mRNA levels by quantitative real-time PCR:**

**GAPDH**

Forward: 5’-GAAGGCTGGGGCTCATTT-3’

Reverse: 5’-CAGGAGGCATTGCTGATGAT-3’

**SLC7A11**

Forward: 5’-TGCTGGGCTGATTTTATCTTCG-3’

Reverse: 5’-GAAAGGGCAACCATGAAGAGG-3’

**SLC3A2**

Forward: 5’-CTGGTGCCGTGGTCATAATC-3’

Reverse: 5’-GCTCAGGTAATCGAGACGCC-3’

**The primer pairs were used for PCR cloning of SLC7A11 or SLC3A2 CDS regions:**

**SLC7A11**

Forward: 5’ -CCGGAATTCACCATGGTCAGAAAGCCTGTTGTGTCC-3’

Reverse: 5’ -TAGTCGACGCAGATTGCCAAGATCTCAAGTCC-3’

**SLC3A2**

Forward:

5’ -TCCTTCTCTAGGCGCCGGCCGGATCCACCATGGAGCTACAGCCTCCT-3’

Reverse:

5’ -TCGACCACTGTGCTGGCGAATTCTCAGGCCGCGTAGGGGAAG -3’

**Primer pairs used for quantitative real-time PCR for SLC7A11 and SLC3A2 after ChIP**

**SLC7A11-ERE**

Forward:5’-GGTAATTCAAAGGAACAACATGAC-3’

Reverse:5’-GTCACATACACACAACTATAAGCC-3’

**SLC3A2-ERE**

Forward:5’-AGACAAAGTACAAAAGAGTAACAGC-3’

Reverse:5’-TACGCCCTCCTTCCCTGGAACT-3’
