# Supplementary material for: Targeting estrogen-regulated system xc− promotes ferroptosis and endocrine sensitivity of ER+ breast cancer
Source: Cell Death Dis. 2025 Jan 20;16(1):30. doi: 10.1038/s41419-025-07354-0 (PMC11756422; doi:10.1038/s41419-025-07354-0)
Supplement: Supplementary file 3 — original data [file 41419_2025_7354_MOESM3_ESM.pdf]

Original images for Western blot

Figure 1C

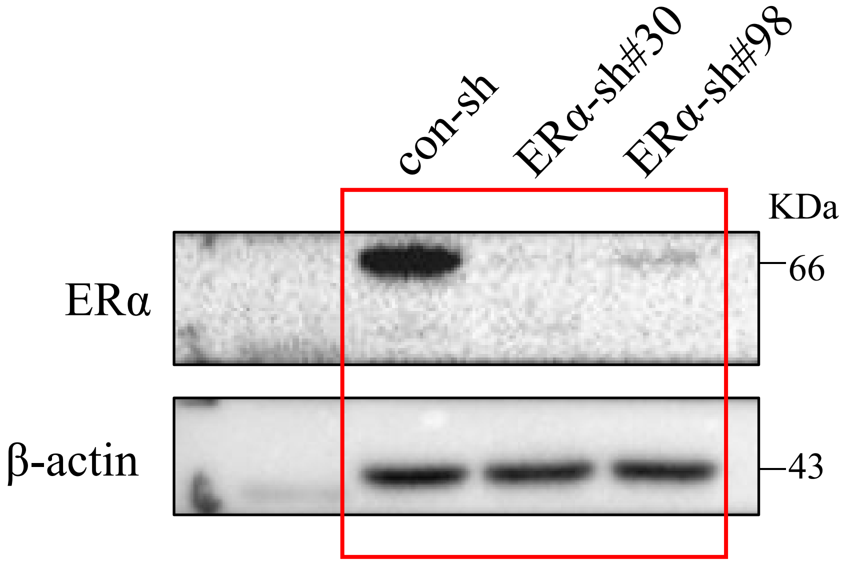

• MCF-7

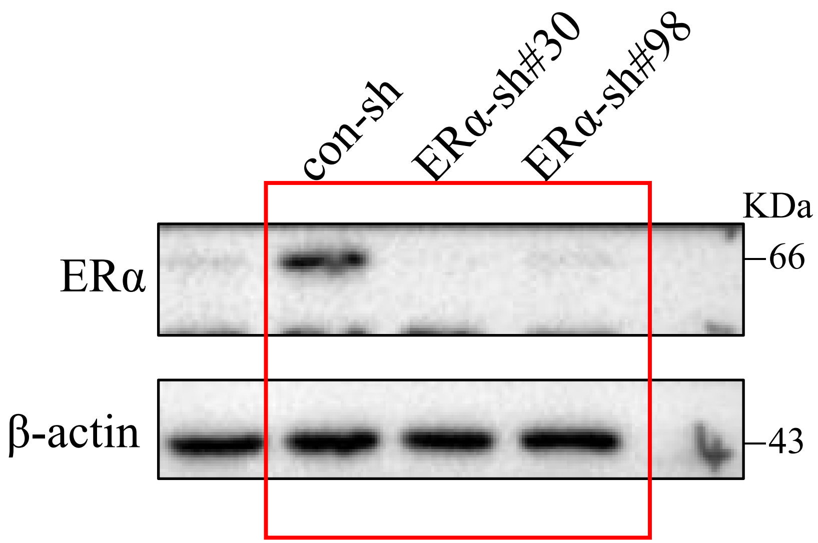

• ZR-75-1

Figure 3C

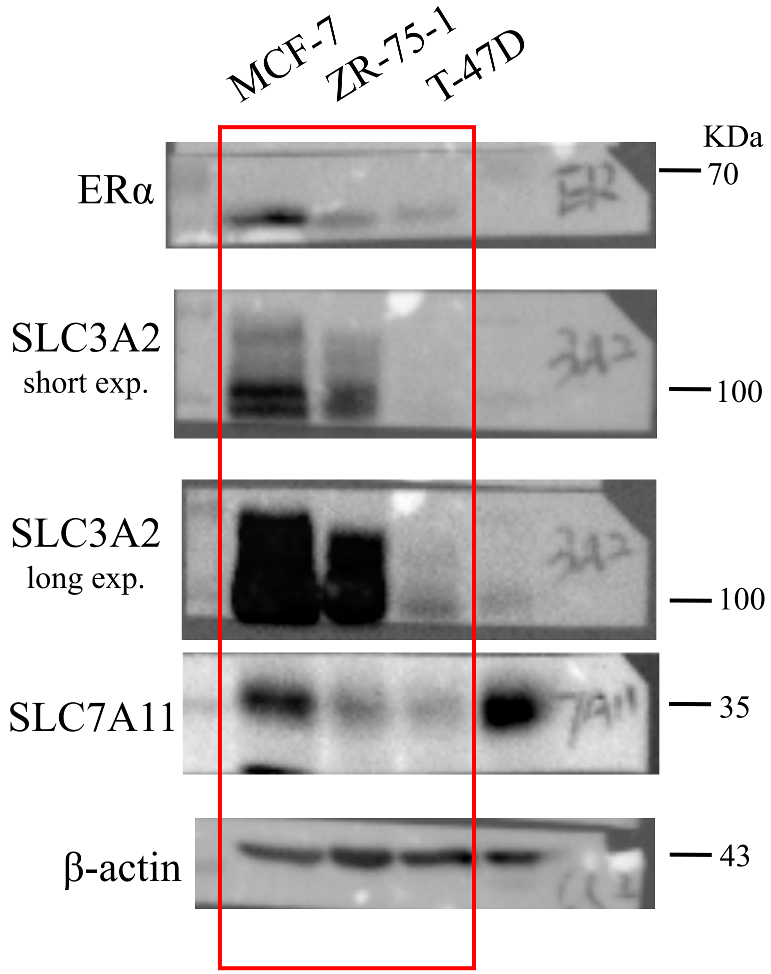
MCF-7
ZR-75-1
T-47D
KDa
ERα
70
SLC3A2
short exp.
100
SLC3A2
long exp.
100
SLC7A11
35
β-actin
43

Figure 4C

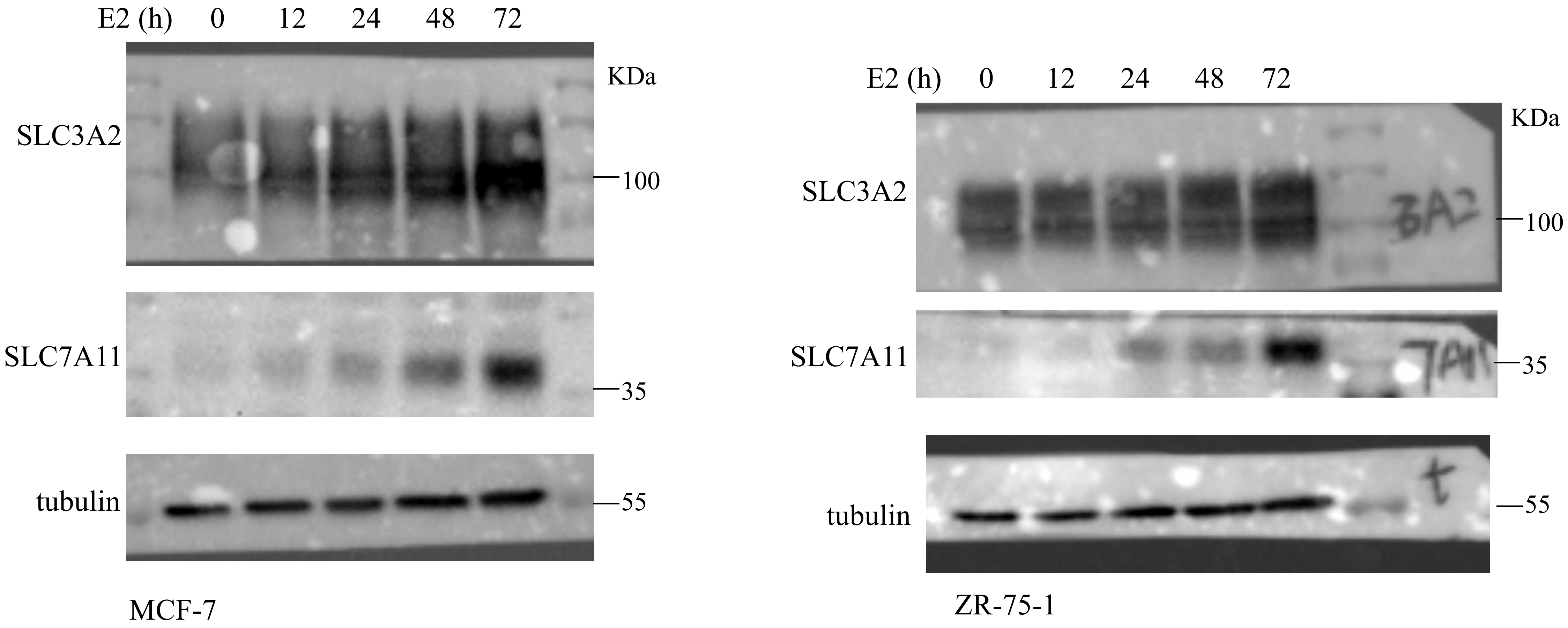
E2 (h) 0 12 24 48 72
KDa
SLC3A2
100
SLC7A11
35
tubulin
55
MCF-7
E2 (h) 0 12 24 48 72
KDa
SLC3A2
100
SLC7A11
35
tubulin
55
ZR-75-1

Figure 4D

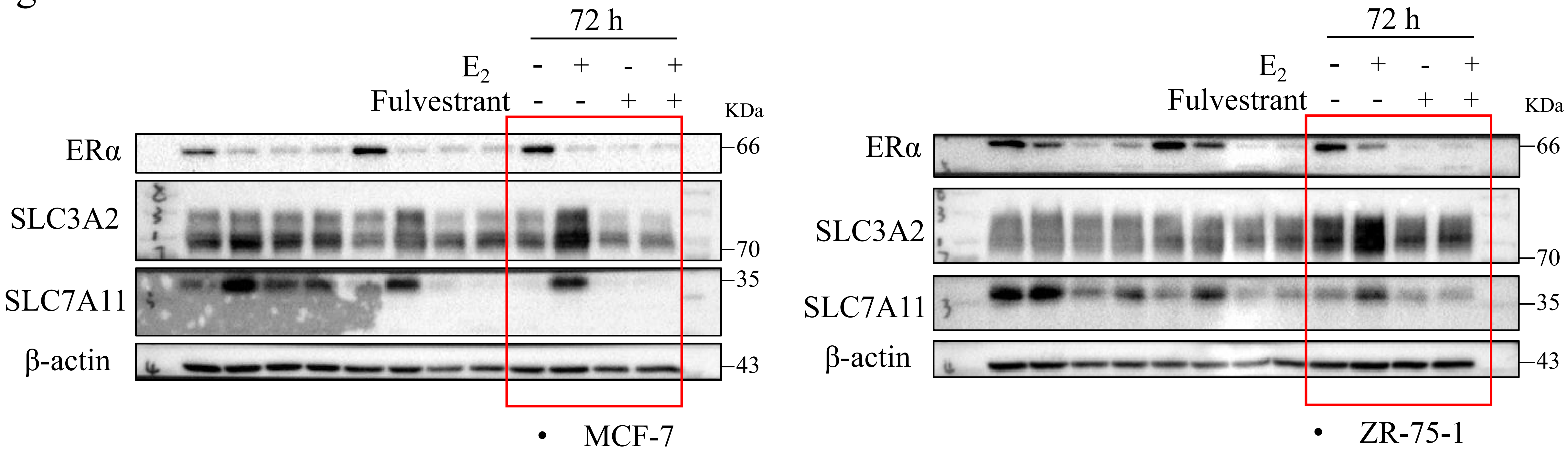

72 h
E2 - + - +
Fulvestrant - - + +
KDa
ERα
SLC3A2
SLC7A11
β-actin
66
70
35
43
• MCF-7
72 h
E2 - + - +
Fulvestrant - - + +
KDa
ERα
SLC3A2
SLC7A11
β-actin
66
70
35
43
• ZR-75-1

Figure 4F

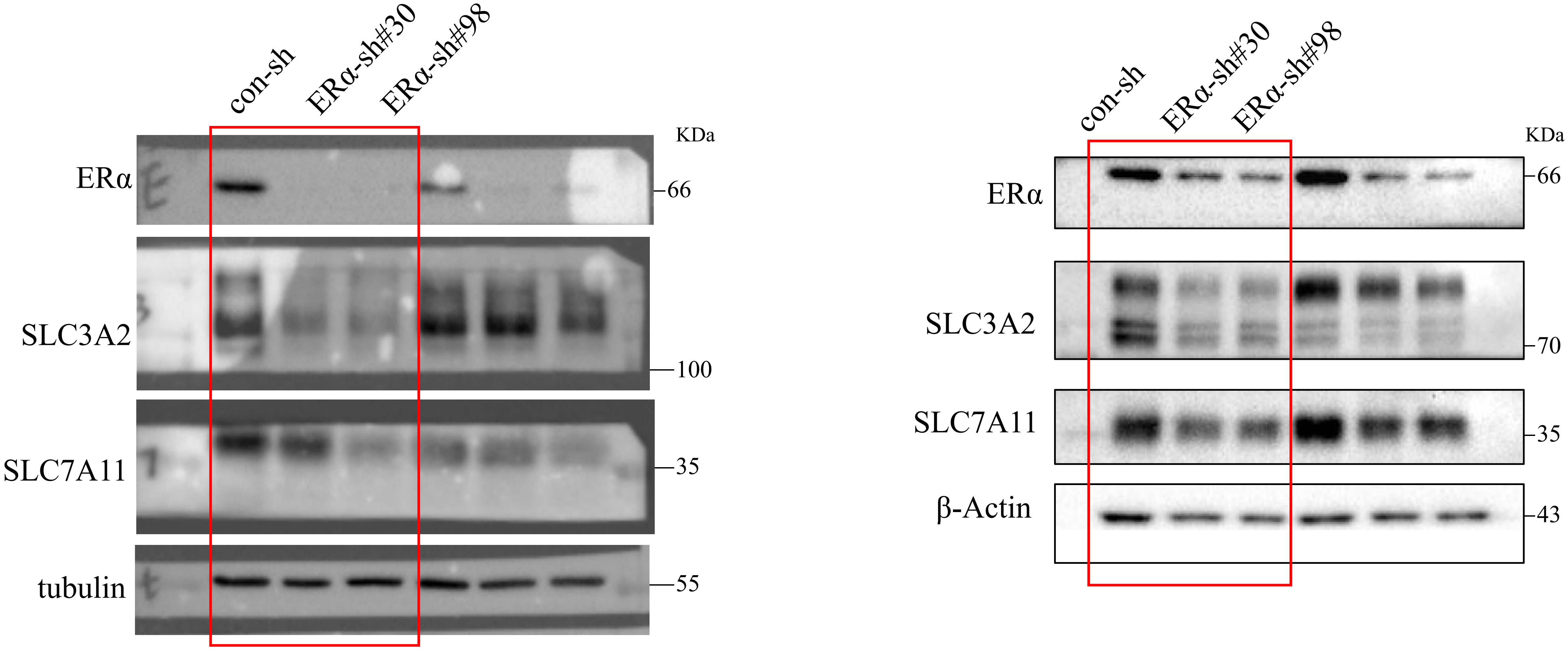

- MCF-7
- ZR-75-1

Figure 5A

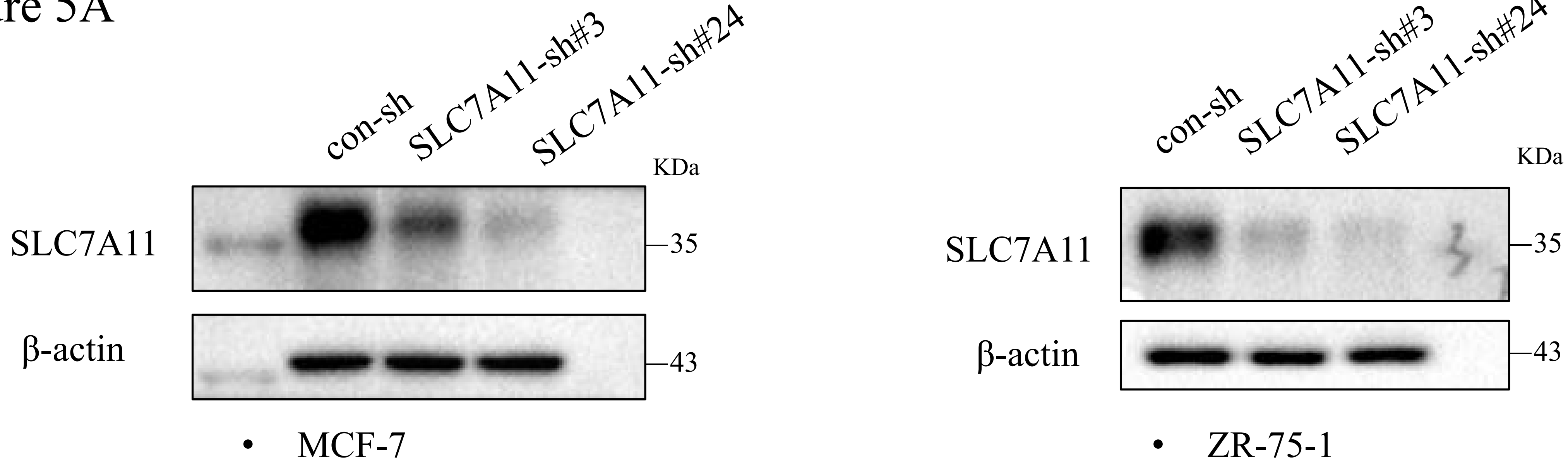

Figure 5B

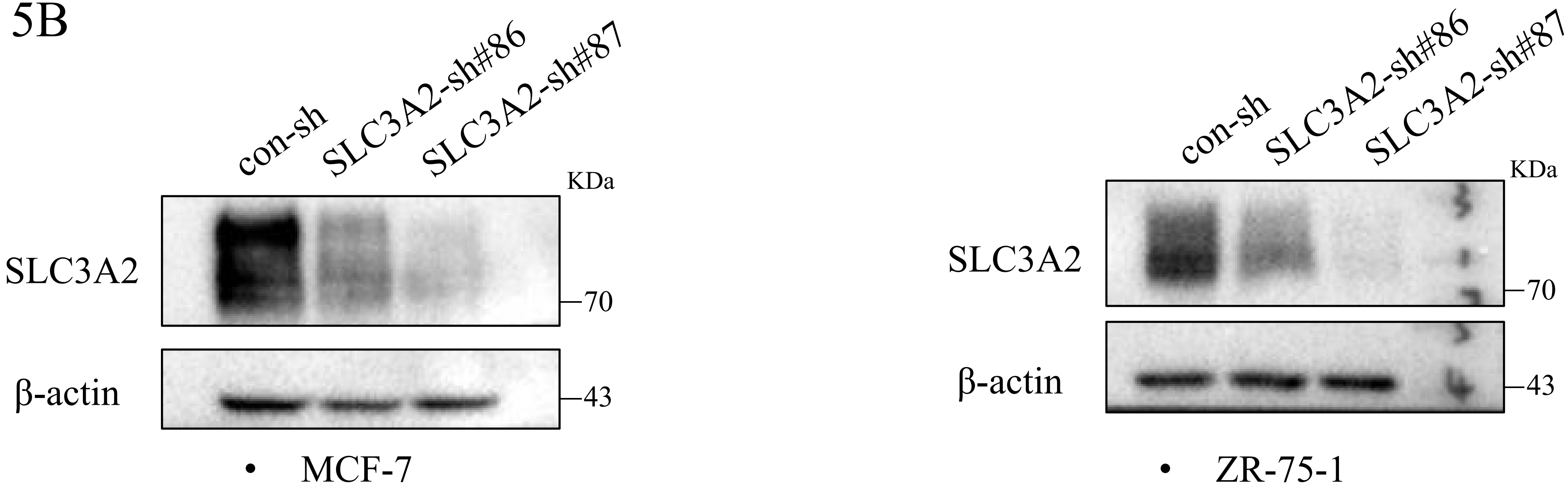

Figure 5F

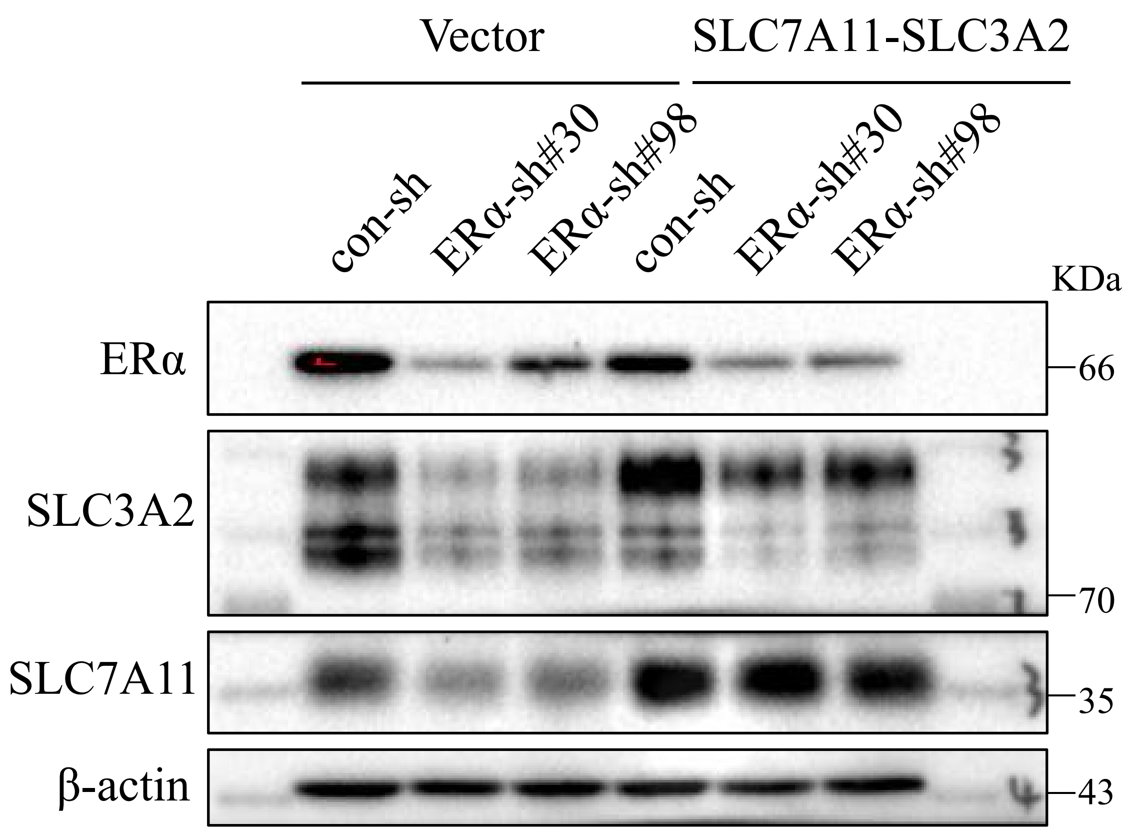

- MCF-7

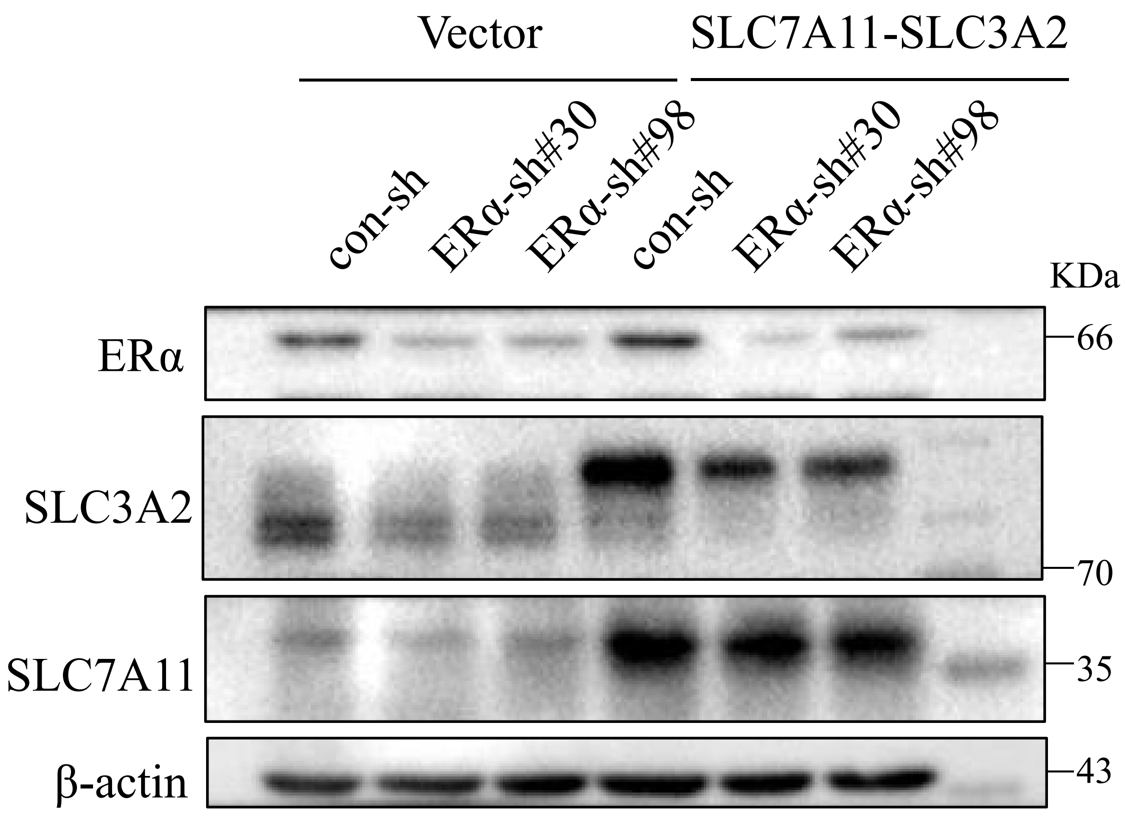

- ZR-75-1

Figure 6B

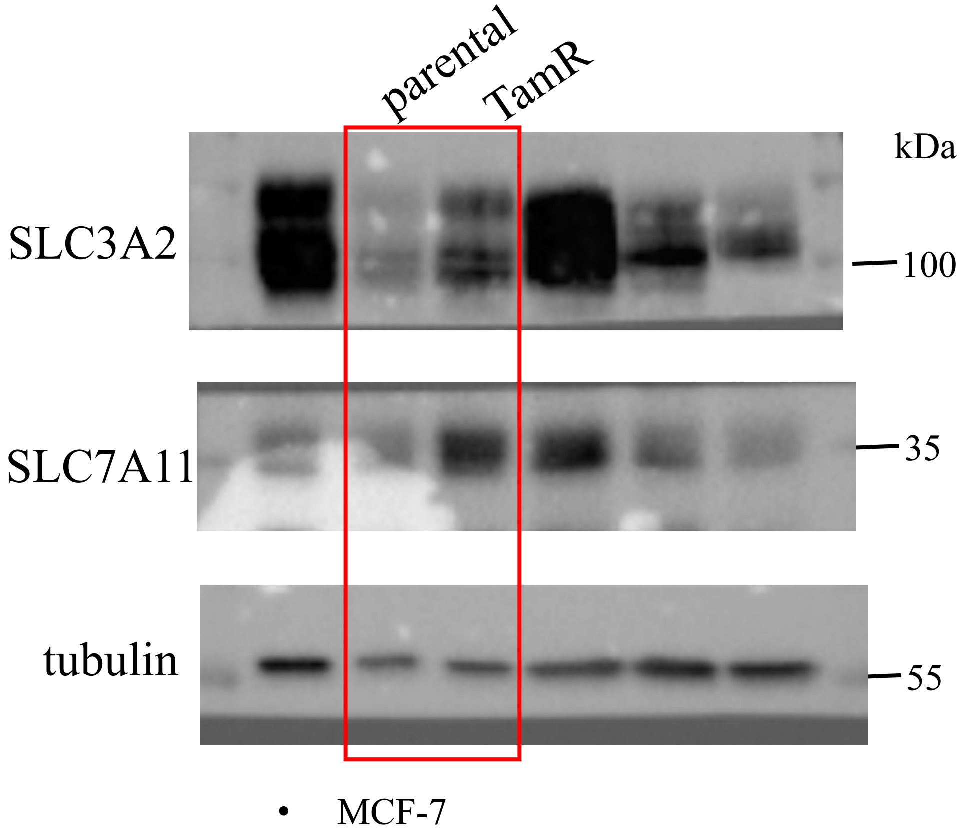

Figure S5A

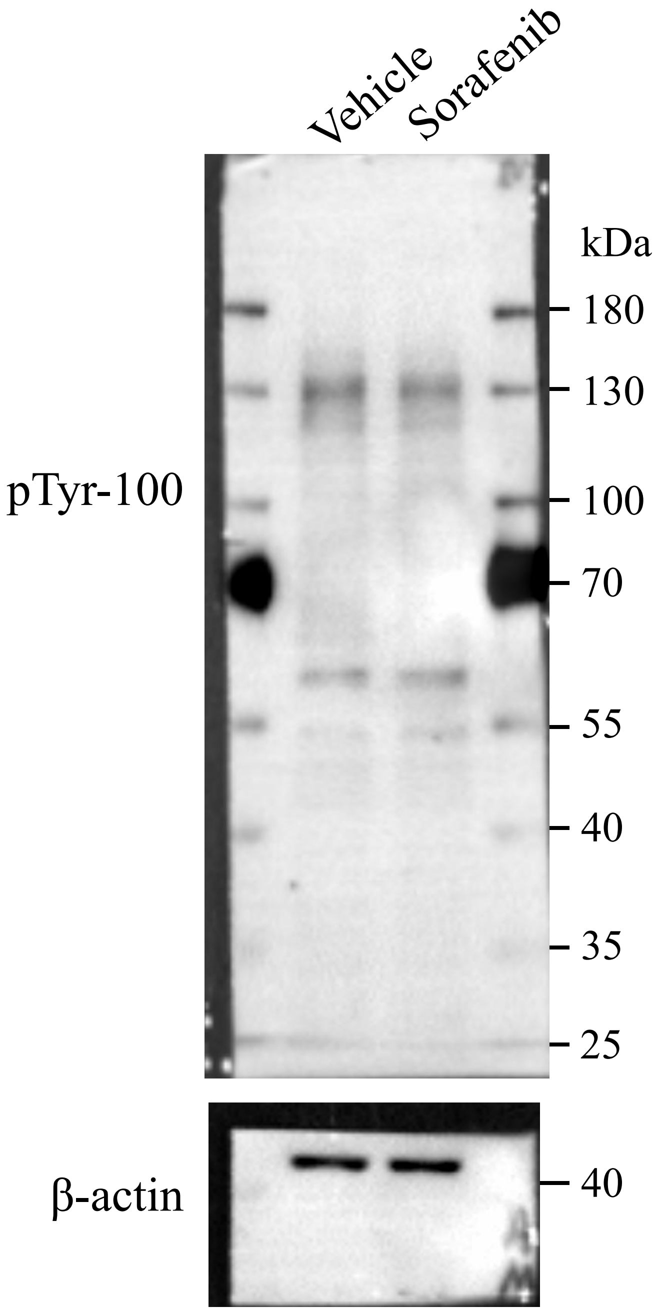

• MCF-7-TamR

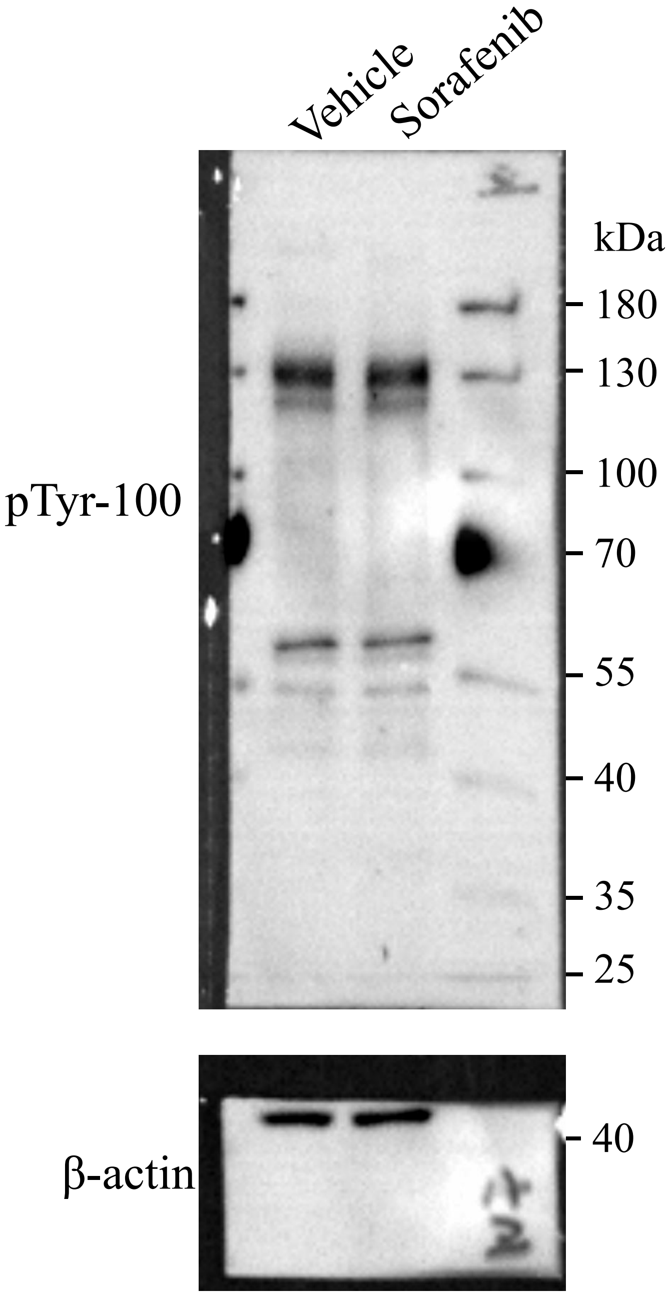

• ZR-75-1-TamR

Figure S5B

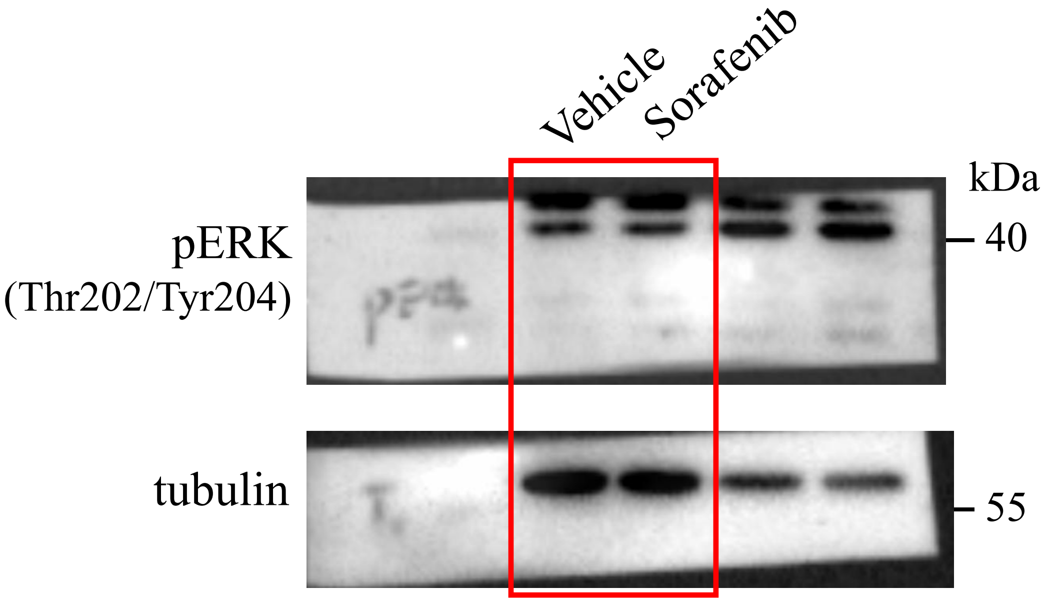

• MCF-7-TamR

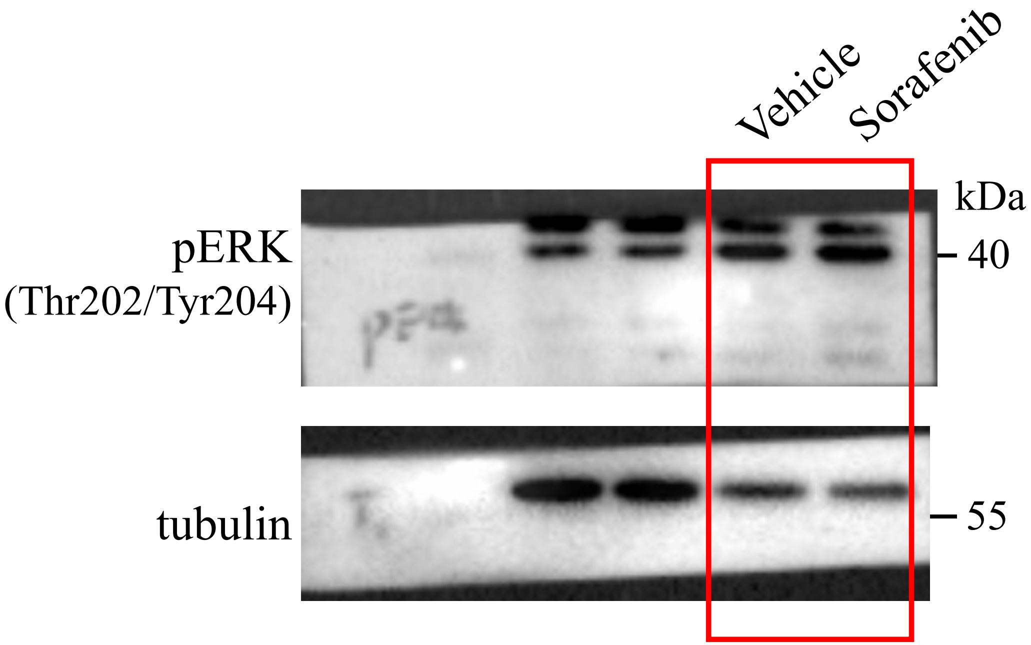

• ZR-75-1-TamR

Original data for q PCR

Figure 4A

MCF-7 SLC7A11				
0	12	24	48	72
0.900539106	17.31554734	8.361365436	24.27790448	44.0254752
0.957746967	19.62600105	7.922584662	24.35981928	41.05922372
1.09401622	23.04818173	8.568825613	25.95646982	44.31209353
1.059796322	20.99680038	8.472635964	26.06597516	46.82307525

ZR-75-1 SLC7A11				
0	12	24	48	72
0.972763359	1.899280076	3.82456401	2.042774228	7.20456457
0.856062401	1.968949059	3.543140847	2.313565699	7.03123151
0.976105492	2.248213403	4.078041711	2.108688168	7.095643269
1.230243632	2.126147262	3.925382136	2.653074085	7.524667982

Figure 4B

MCF-7 SLC3A2				
0	12	24	48	72
0.959531666	5.250445518	3.982034783	13.2079074	18.65068481
0.965267789	4.847231995	4.03061464	12.63382443	18.32084354
1.033520531	4.904744004	2.725265513	12.68882917	18.61055859
1.044657171	5.349175365	4.219975921	13.45632607	19.35646611

ZR-75-1 SLC3A2				
0	12	24	48	72
0.946914512	1.314086088	1.87456112	1.858284325	2.298873689
0.973584174	1.306265228	1.728490759	1.844479198	2.584920849
1.039304412	1.297845245	1.89706063	1.881747903	2.357938811
1.043693517	1.267956927	1.997589325	1.822770679	2.431545454

Figure 4E

MCF-7 SLC7A11			MCF-7 SLC3A2		
con-sh	ERα-sh#30	ERα-sh#98	con-sh	ERα-sh#30	ERα-sh#98
0.895533869	0.418206831	0.326919573	1.081857148	0.412790852	0.376773706
1.065676966	0.560503552	0.323173125	0.898543262	0.367874017	0.374148776
0.930908426	0.675264967	0.333403321	0.95386235	0.47510788	0.362036705
1.125605025	0.586585282	0.366499101	1.078463466	0.463460698	0.329549118

ZR-75-1 SLC7A11			ZR-75-1 SLC3A2		
con-sh	ERα-sh#30	ERα-sh#98	con-sh	ERα-sh#30	ERα-sh#98
0.837281084	0.390601476	0.380909419	1.038767381	0.652661873	0.596261425
0.938798442	0.350518034	0.38825753	0.984648979	0.682866919	0.539596122
1.165912032	0.477043128	0.460175987	0.99252501	0.685965829	0.59345214
1.091164319	0.542961382	0.547848147	0.985051092	0.709961077	0.520610379

Figure 4L

SLC7A11						
	IgG			ERα		
vehicle	2.48410296	2.176684692	0.184941721	1.303431774	0.986456565	0.760469981
E_2	0.363031824	0.8856803	1.475605262	15.45864645	17.24029103	11.87979339

SLC3A2						
	IgG			ERα		
Vehicle	1.110668868	0.277207501	1.605660792	3.167375996	0.691520274	0.188389497
E_2	1.5494814	0.477857948	0.621813646	8.141365348	8.365777868	8.94864544
